# IL-33/ST2 Signaling Protects the Heart by Restraining Inflammation and Parasite Burden during *Trypanosoma cruzi* Experimental Infection

**DOI:** 10.1021/acsinfecdis.5c00859

**Published:** 2025-12-09

**Authors:** Marcelo Eduardo Cardozo, Tatyane Martins Cirilo, Jorge Lucas Nascimento Souza, José Bryan da Rocha Rihs, Isabela de Brito Duval, Fernando Bento Rodrigues Oliveira, Mayra Ricci, Laura Lis de Oliveira Santos, Livia Fernanda Santana, Luiza Pinheiro Silva, Chiara Cassia Oliveira Amorim, Ana Rafaela Antunes-Porto, Izabela da Silva Oliveira, Ana Laura Grossi de Oliveira, Luisa Vitor Braga do Amaral, Gabriela Gomes Monteiro Lemos, Getúlio Mota e Silva Junior, Ivan Lobo de Sousa Marques, Marina Possa dos Reys, Geovanni Dantas Cassali, Artur Santos-Miranda, Luisa Mourão Dias Magalhães, Lilian Lacerda Bueno, Fabiana Simão Machado, Ricardo Toshio Fujiwara

**Affiliations:** † Laboratory of Immunobiology and Parasite Control, Institute of Biological Sciences, 28114 Universidade Federal de Minas Gerais, Belo Horizonte 31270-901, Brazil; ‡ Post-graduation Program in Health Sciences: Infectious Diseases and Tropical Medicine, Faculdade de Medicina, Universidade Federal de Minas Gerais, Belo Horizonte 30130-100, Brazil; § Laboratory of Immunoregulation of Infectious Diseases, Institute of Biological Sciences, Universidade Federal de Minas Gerais, Belo Horizonte 31270-901, Brazil; ∥ Laboratory of Cellular Electrophysiology, Institute of Biological Sciences, Universidade Federal de Minas Gerais, Belo Horizonte 31270-901, Brazil; ⊥ Laboratory of Comparative Pathology, Institute of Biological Sciences, Universidade Federal de Minas Gerais, Belo Horizonte 31270-901, Brazil; # Laboratory of Interactions in ImmunoParasitology, Institute of Biological Sciences, Universidade Federal de Minas Gerais, Belo Horizonte 31270-901, Brazil; ¶ Institute of Research in Mucosa and Skin (INCT Mucosa and Skin), Belo Horizonte 31270-901, Brazil

**Keywords:** Chagas disease, myocarditis, inflammation, cytokines, leukocytes

## Abstract

Chagas disease (CD), caused by the parasite *Trypanosoma cruzi*, affects millions of people worldwide and often leads to fatal heart damage. The course of CD is influenced by how the immune system is tuned, which can protect the host and favor parasite persistence. Interleukin-33 (IL-33) is an alarmin released upon tissue injury that signals through the ST2 receptor, exerting context-dependent regulatory or pathogenic effects. However, the role of the IL-33/ST2 axis in *T. cruzi*-induced myocarditis remains unclear. Here, using ST2-deficient (ST2^–/–^) and wild-type (WT) female mice infected with the *T. cruzi* Y strain, we investigated its contribution to cardiac inflammation, tissue damage, and parasite burden. We found that ST2 signaling was not essential for controlling parasitemia, but its deficiency led to early onset myocarditis, disorganized fibrosis, and distinct electrical conduction. This severe immunopathology was driven by a remodeling of the cardiac immune landscape, with ST2^–/–^ mice exhibiting an influx of IFN-γ-producing monocytes and a shift in resident and monocyte-derived macrophages toward a pathogenic, pro-inflammatory phenotype. Likewise, the cardiac T cell compartment, including both conventional and γδ T cells, was skewed toward an inflammatory, IFN-γ-driven profile. However, infected ST2-deficient mice also displayed higher cardiac parasite burden and impaired nitric oxide production, indicating a dysfunctional response in parasite control. Together, these findings demonstrate that the IL-33/ST2 axis limits early systemic inflammation, orchestrates cardiac immune response, and protects against immunopathology and electrical remodeling during *T. cruzi* experimental infection. Targeting this pathway may offer therapeutic potential for preventing cardiac damage in CD.

## Graphical abstract

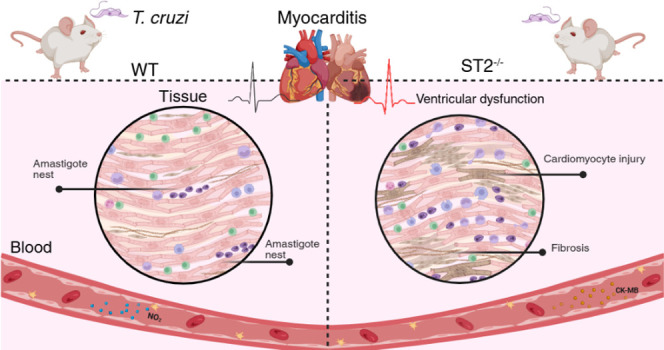

1

Chagas disease (CD) is a neglected tropical disease caused by the protozoan parasite *Trypanosoma cruzi*. Approximately 6–7 million people are infected worldwide, primarily in Latin America, where CD imposes a major socioeconomic burden.^1,2^ Due to human migration, the disease has also become a global health concern. Transmission occurs mainly through blood-feeding reduviid insects, but alternative routes, such as oral outbreaks, have been increasingly recognized. Early diagnosis is uncommon, and effective, nontoxic treatments remain scarce, particularly during the chronic stage.^2–6^

Following infection, *T. cruzi* disseminates widely, but the heart is one of the main targets. Chagasic cardiomyopathy (CCC) develops in ∼30% of patients and represents the most severe clinical outcome, characterized by persistent myocarditis, fibrosis, and cardiac remodeling that lead to arrhythmias, heart failure, and sudden death.^7–9^ Disease progression depends on a combination of parasite persistence, host genetics, and characteristics of the immune response.^10,11^

A strong type 1 immune response, with inflammatory cytokines, nitric oxide, and cytotoxic mechanisms, is essential for parasite control.^12,13^ However, the insufficient regulation of these responses drives tissue damage. Indeed, CCC patients exhibit high levels of proinflammatory cytokines, like TNF-α and IFN-γ, whereas individuals with asymptomatic disease show higher IL-10, highlighting the central role of immune balance.^14,15^ In this context, the IL-33/ST2 pathway has emerged as a key damage-sensing immune axis. IL-33 is released upon tissue damage and signals through the receptor ST2, activating MyD88-dependent cascades that modulate both Th1 and Th2 responses.^16–18^

The IL-33/ST2 axis has been implicated in host–parasite interactions during *T. cruzi* infection. In vitro, IL-33 stimulation of J774 macrophage-like cells enhances TNF, IL-17, and CCL2 production while reducing intracellular parasite burden.^19^ Moreover, cells from patients with CD across different clinical forms display IL-33 expression, highlighting a potential role for this pathway in parasite-induced inflammation.^20^ More recently, Boccardo and colleagues^21^ elegantly demonstrated that the progression of *T. cruzi* infection is marked by a decline in IL-33 levels. In the same study, they showed that early IL-33 supplementation sustains tissue-repair regulatory T cell dynamics and protects against skeletal muscle damage. However, the role of the endogenous IL-33/ST2 axis in the progression from the acute to chronic phase, particularly in the context of cardiac pathophysiology, has not been elucidated.

Here, we investigated the role of the IL-33/ST2 axis in the development of myocarditis during acute and chronic *T. cruzi* infection. Using ST2-deficient mice, we evaluated how this pathway regulates cardiac inflammation, fibrosis, function, and parasite control. Understanding these mechanisms may help explain the clinical heterogeneity of CD and inform new therapeutic strategies to mitigate cardiac disease.

## 2 Results

### 2.1 The IL-33/ST2 Axis Regulates Early Systemic Leukocyte Dynamics during *T. cruzi* Infection

To investigate the role of the IL-33/ST2 pathway in host defense against *T. cruzi*, we first characterized the systemic infection kinetics in ST2-deficient (ST2^–/–^) and wild-type (WT) BALB/c mice. Following intraperitoneal infection, we monitored parasitemia and survival for 100 days. Both groups exhibited a similar overall pattern of parasitemia, with a peak at 9 days post-infection (dpi) and subsequent clearance (Figure 1A). Notably, ST2 KO mice displayed a reduction in parasitemia at 7 dpi compared to WT mice (Figure S1). However, parasitemia levels converged by 9 dpi, leading to similar kinetics with no detectable parasites in the blood by 30 dpi. Although not statistically significant, ST2^–/–^ mice exhibited a trend toward earlier mortality, with deaths beginning at 17 dpi compared to 29 dpi in WT animals (Figure 1B).

**Figure 1:**
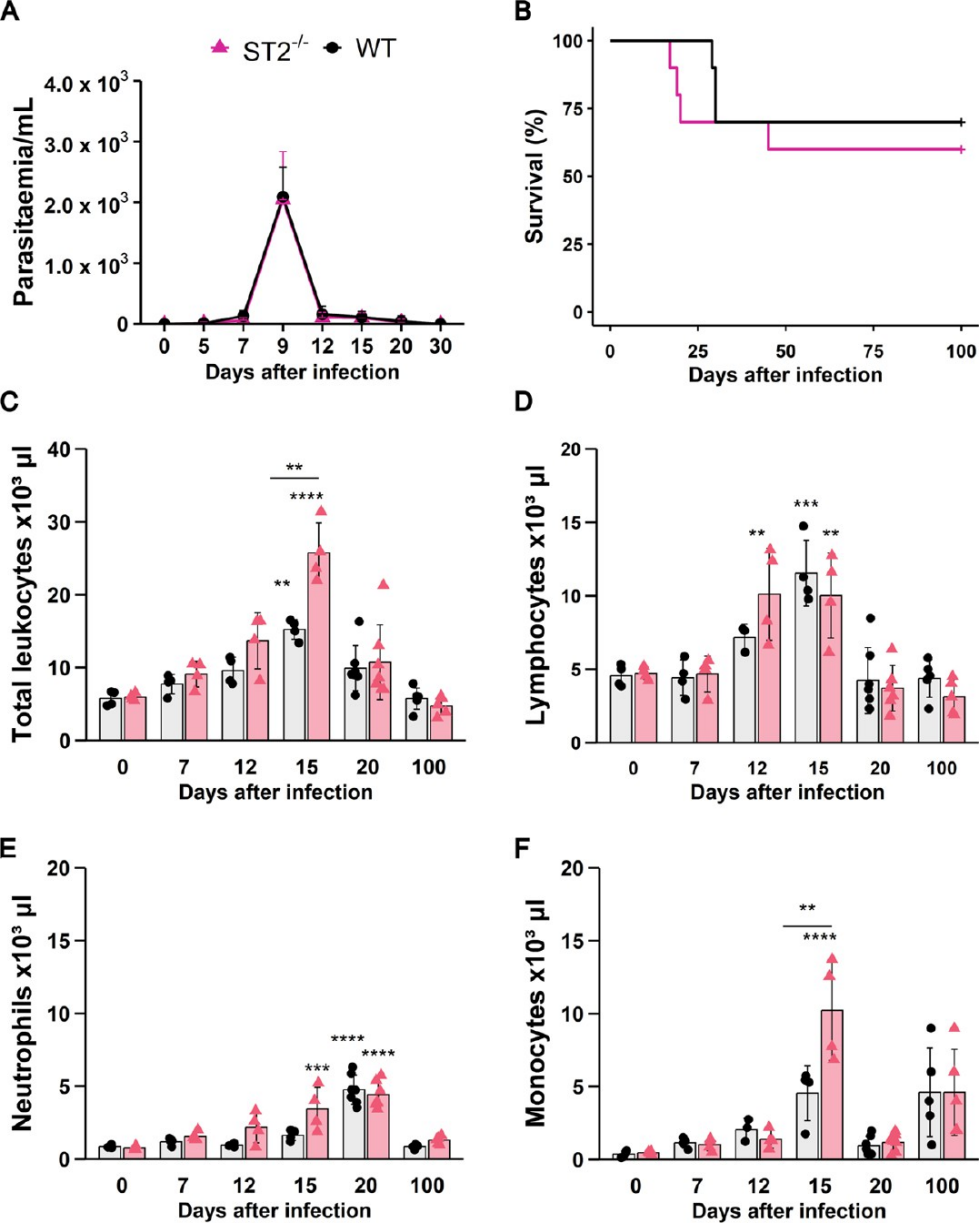
Blood parasitism and leukocyte profile during *T. cruzi* infection in WT and ST2^–/–^ mice. WT and ST2^–/–^ mice were infected with 1000 *T. cruzi* blood trypomastigotes and monitored for up to 100 days, during acute and chronic phases of infection. (A) Number of parasites per mL of blood. (B) Survival rate. (CF) Kinetic analysis of circulating leukocyte populations, including (C) total leukocytes, (D) lymphocytes, (E) neutrophils, and (F) monocytes. *N* = 4–8 biological replicates per group from two independent experiments. Results are represented by the mean ± SD **p < 0.01, ***p < 0.001, ****p < 0.0001, represented by the asterisk when each strain is compared with its respective noninfected group (0 dpi), and *p*-values with the bar represent the comparison between both strains at the same time of infection. Time series data were analyzed using two-way ANOVA with Tukey post hoc testing. WT: wild-type.

Because circulating leukocytes play a critical role in shaping antiparasitic immunity, we next examined blood cell dynamics during acute and chronic (100 dpi) phases of infection (Figure 1C–F). In this experimental model, peak tissue inflammation is typically observed around 20 dpi.^22^ We hypothesized that critical shifts in circulating leukocyte populations might precede this tissue-level response. Therefore, to better capture the dynamic leukocyte response, our analysis included earlier time points (7, 12, and 15 dpi). The rationale for these additional points also stems from the early reduction in parasitemia at 7 dpi in KO mice, suggesting that immune alterations may already be occurring. WT mice developed a robust leukocytosis that peaked at 15 dpi (Figure 1C). However, this response was more pronounced in ST2^–/–^ mice. The KO group exhibited an earlier onset of systemic inflammation, with significantly elevated numbers of lymphocytes and neutrophils already present at 12 and 15 dpi, respectively, when compared to naive controls (0 dpi) (Figure 1D,E). By 15 dpi, the leukocytosis in ST2^–/–^ mice was predominantly composed of monocytes, which were markedly increased compared to both their uninfected controls and the infected WT group (Figure 1F). By 20 dpi, circulating leukocyte counts had decreased in both groups, suggesting recruitment to tissues.

Together, these findings indicate that IL-33/ST2 signaling modulates the early systemic leukocyte response during acute *T. cruzi* infection, leading us to investigate whether these early systemic changes translate into alterations in cardiac pathology.

### 2.2 IL-33/ST2 Signaling Limits Early Myocardial Inflammation and Fibrotic Remodeling during *T. cruzi* Infection

The heart is a major target organ during *T. cruzi* infection, and myocarditis is a central pathological feature of experimental CD models. Based on the accelerated systemic inflammation that we observed in ST2^–/–^ mice (Figure 1), we hypothesized that cardiac damage might also be occurring earlier in these animals. To test this, we performed a kinetic histopathological analysis of the heart at early acute (7 dpi), peak acute (20 dpi), and chronic (100 dpi) phases. To further characterize histopathological changes, we used an established quantitative grading system to evaluate the severity of myocarditis (Table 1).^23^

**Table 1:** Histological Grading Criteria

grade	description
0	no inflammatory infiltrates
1	limited focal distribution of inflammatory infiltrates
2	multiple lesions with inflammatory infiltrates
3	multiple lesions with confluence and some extension
4	lesions affecting most of the observed tissue

At 7 dpi, WT-infected mice displayed minimal or absent inflammatory foci, whereas ST2^–/–^ mice already exhibited moderate myocarditis, as reflected in significantly higher inflammation scores (Figure 2A,B). This early histopathological damage was corroborated by significantly elevated serum levels of the cardiac injury marker creatine kinase-MB (CK-MB) in ST2^–/–^ mice at 7 dpi (Figure 2C). By 20 dpi, the peak of acute inflammation, both groups displayed severe and extensive myocarditis. However, the lesions in ST2^–/–^ featured prominent areas of cardiomyocyte degeneration (indicated by stars, Figure 2A) and persistently higher CK-MB levels (Figure 2C). In the chronic phase (100 dpi), inflammation subsided in both groups but remained multifocally distributed in the hearts of ST2^–/–^ mice, along with residual areas of cardiomyocyte degeneration, as indicated by the stars in Figure 2B. Despite these differences in immunopathology, we observed no significant changes in heart weight or the number of amastigote nests between the groups at any time point (Figure S2).

**Figure 2:**
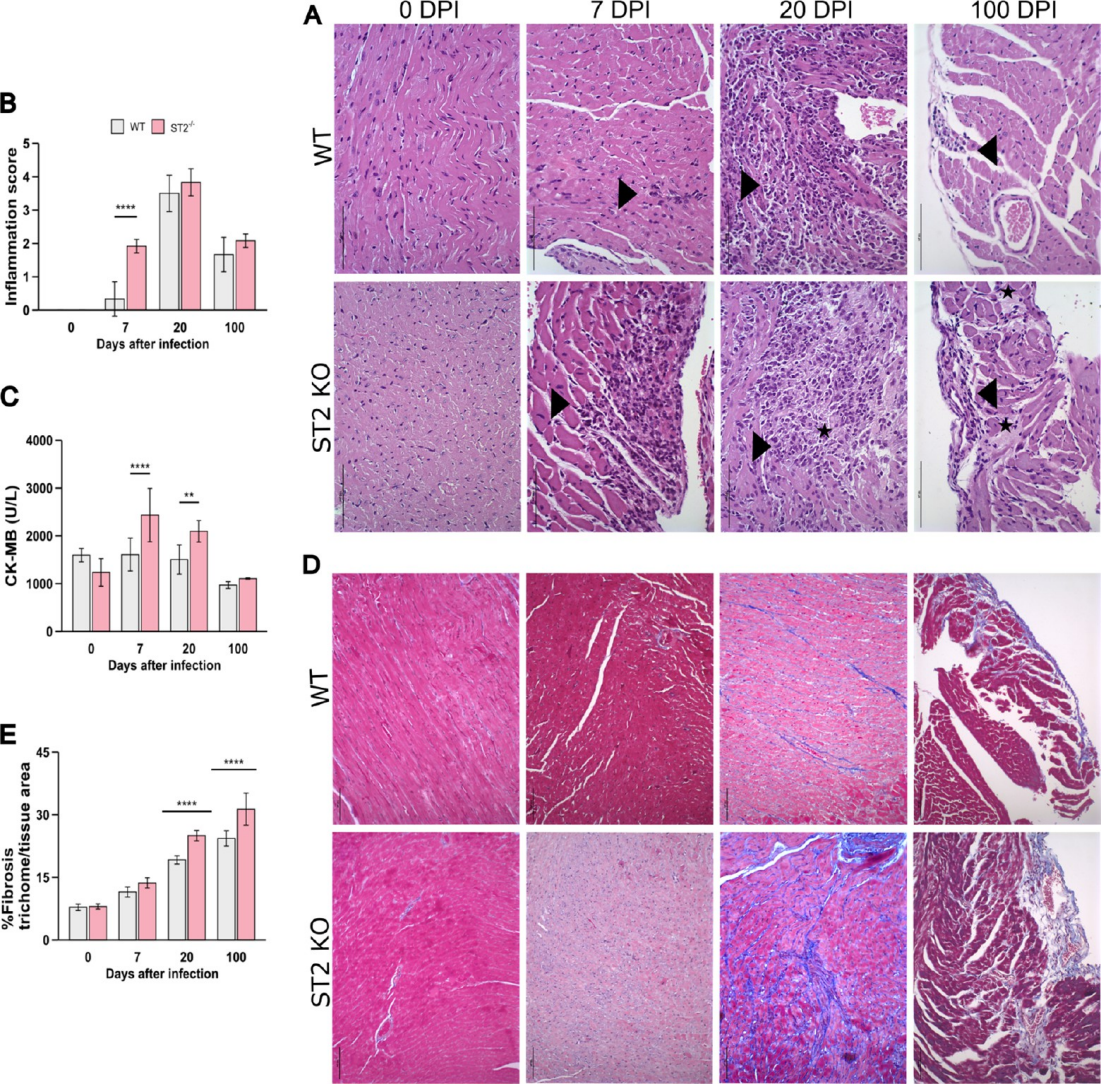
Characterization of heart inflammation, damage, and fibrosis in ST2^–/–^ and WT mice infected with *T. cruzi*. Hematoxylin and eosin (H&E) was used to evaluate inflammation, and the collagen deposition area was measured by Masson’s trichome at the acute (7, 20 dpi) and chronic phases (100 dpi) of infection. (A) Heart histological sections after coloration with HE and representative of WT and ST2^–/–^ mice noninfected and infected with *T. cruzi*. Arrows represent inflammatory foci and stars represent degenerative lesions. Scale bar: 100 μm. (B) Semiquantitative inflammation analysis by the score of heart inflammation. (C) Measurement of serum creatine kinase-MB (CK-MB) levels. (D) Representative images of heart sections stained with Masson’s trichrome to visualize collagen deposition (blue). Scale bar: 100 μm. (E) Percentage of cardiac fibrosis quantified by ImageJ software version 1.54p. *N* = 6 biological replicates per group. Results are represented by the mean ± SD **p < 0.01, ***p < 0.001, ****p < 0.0001, represented by the asterisk when each strain is compared with its respective noninfected group (0 dpi), and *p*-values with the bar represent the comparison between both strains at the same time of infection. Time series data were analyzed using two-way ANOVA with Tukey post hoc testing. WT: wild-type.

Given that tissue inflammation is a key driver of fibrotic remodeling, we next evaluated collagen deposition using Masson’s trichrome staining. While no significant fibrosis was detected at 7 dpi in either group, by 20 dpi, ST2^–/–^ mice exhibited a more extensive and disorganized pattern of collagen deposition compared to the more limited fibrosis in WT mice (Figure 2D,E). This difference became even more pronounced in the chronic phase. As already described, experimental *T. cruzi*-induced fibrosis in WT mice is often confined to the epicardium.^24^ In contrast, ST2^–/–^ mice displayed a greater total fibrotic area, characterized by extensive interstitial fibrosis, with collagen deposits extending deep into the myocardium.

Taken together, these data demonstrate that the absence of ST2 signaling leads to an earlier and more severe myocarditis, which resolves into a chronic pathology characterized by persistent tissue disturbances and disorganized cardiac fibrosis. This profound alteration of the cardiac architecture, particularly the development of extensive interstitial fibrosis, suggests a potential substrate for electrical conduction abnormalities.

### 2.3 IL-33/ST2 Signaling Regulates Cardiac Parasitism and Nitric Oxide Production during *T. cruzi* Infection

Histopathological analysis revealed different patterns of cardiac parasitism between WT and ST2^–/–^ mice. At 20 dpi, the only time point at which amastigote nests were microscopically detectable, WT mice exhibited large, well-defined nests organized within cardiomyocytes. In contrast, ST2^–/–^ mice displayed more dispersed and fragmented nests, often scattered with areas of degenerating myocardium (Figure 3A). This disorganized presentation in ST2^–/–^ hearts made accurate microscopic quantification of individual nests unreliable (Figure S2).

**Figure 3:**
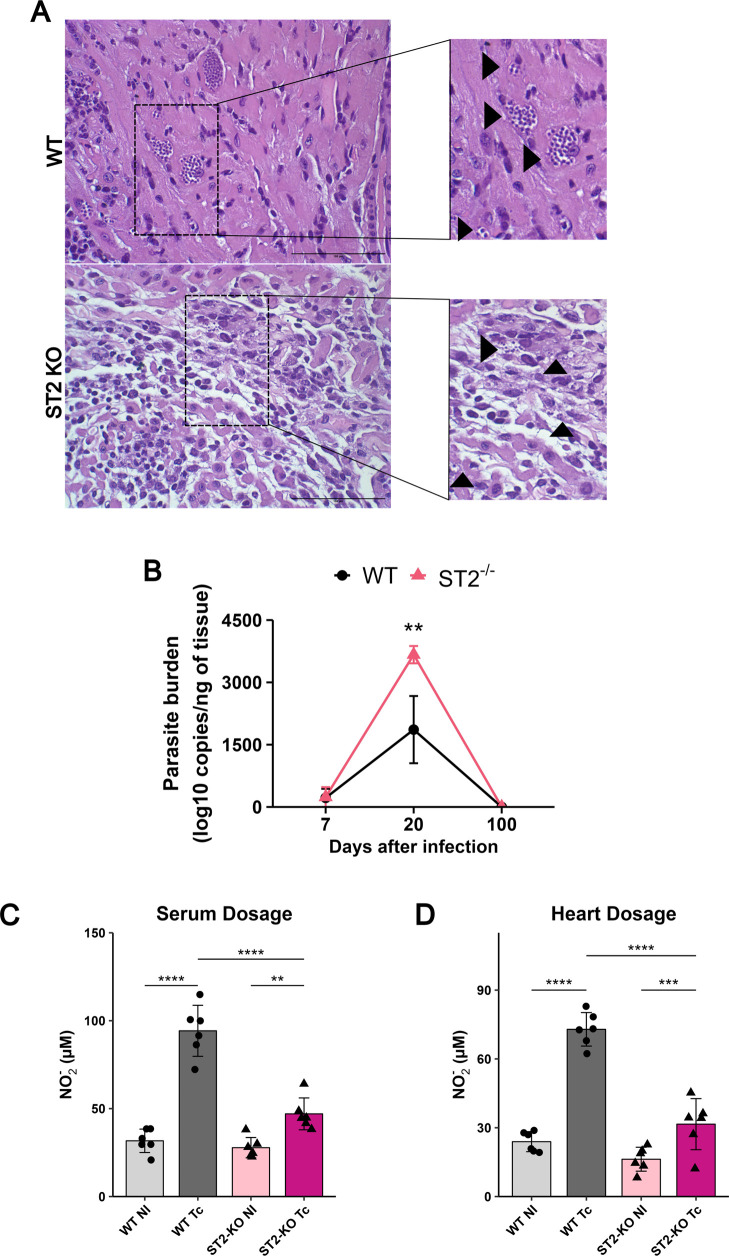
IL-33/ST2 signaling controls heart parasitism and nitric oxide production during *T. cruzi* infection. (A) Representative H&E-stained heart sections at 20 dpi. Black arrowheads indicate amastigote nests. Scale bar: 100 μm. (B) Parasite burden in heart tissue determined by qPCR at 7, 20, and 100 dpi. (C) Nitrite concentration in the serum at 20 dpi, measured by the Griess assay as an indicator of systemic NO production. (D) Nitrite concentration in heart tissue homogenates at 20 dpi. Data are mean ± SD (*n* = 5–6 per group). *p* < 0.01 (one-way ANOVA with the Tukey post hoc test). WT: wild-type.

Therefore, to precisely determine the parasite burden, we quantified parasite DNA using qPCR. Unexpectedly, at 20 dpi, ST2^–/–^ mice exhibited significantly higher parasite numbers in the heart at 20 dpi compared to WT-infected mice (Figure 3B). During the chronic phase, parasite DNA levels declined in both groups, but ST2^–/–^ mice maintained a higher mean parasite burden, although without statistical significance (Table S1). Since nitric oxide (NO) is a primary effector molecule for controlling the replication of intracellular amastigotes, we next quantified nitrite (NO_2_^–^), an indicator of NO production, using the Griess assay. At 20 dpi, KO-infected mice showed reduced nitrite levels in both serum and heart homogenates compared to infected WT mice (Figure 3C,D). Collectively, these data indicate that the IL-33/ST2 pathway plays a crucial role in orchestrating an effective local antiparasitic response in the heart, partly by promoting NO production to control parasite replication.

### 2.4 The IL-33/ST2 Axis is Required to Restrain Pathological IFN-γ T Cell Responses during *T. cruzi*-Induced Myocarditis

After determining the impact of ST2 deficiency on tissue parasitism and damage, we next characterized the immune response at the peak of heart inflammation (20 dpi). To this end, we evaluated whether the absence of the ST2 receptor affects hematopoietic cell recruitment and leukocyte composition within the heart during *T. cruzi* infection by spectral flow cytometry (Figure 4). Immune cells were gated as shown in Figure S3.

**Figure 4:**
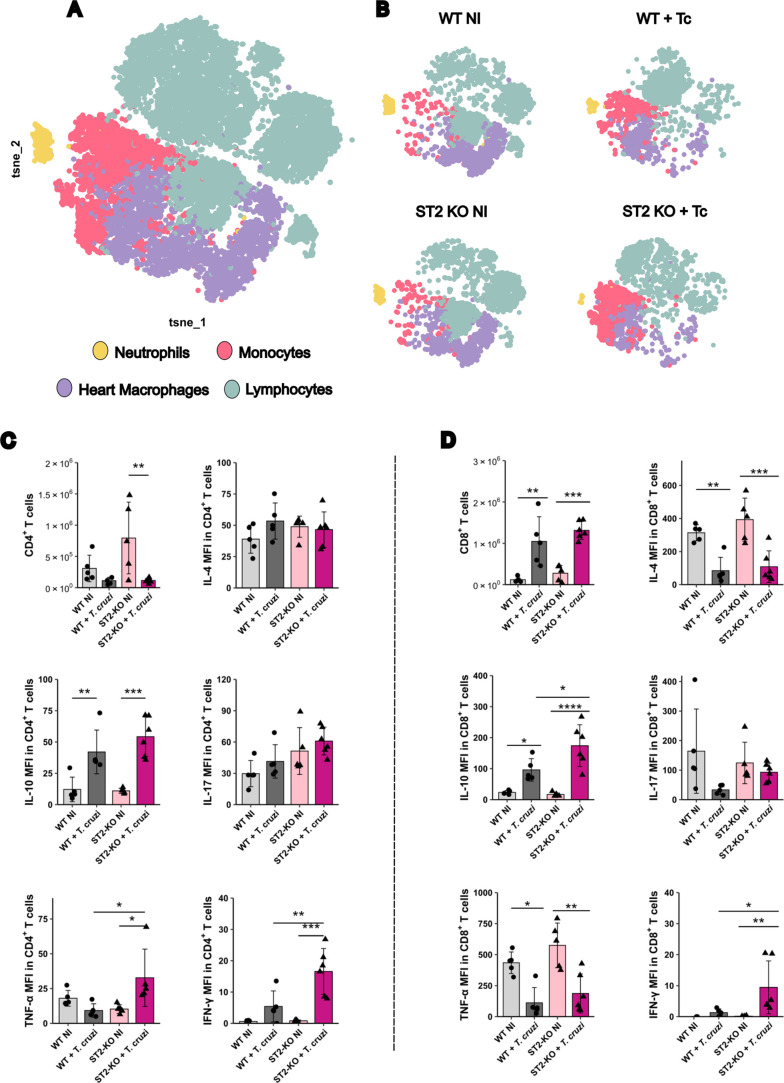
Analysis of the cellular panel and T cell cytokine profile in heart tissue by flow cytometry in WT and ST2^–/–^ mice infected with *T. cruzi* at 20 dpi. (A) Global unsupervised t-SNE analysis showed populations of neutrophils (yellow), monocytes (pink), heart macrophages (purple), and lymphocytes (blue) from all groups and concatenated experimental times. (B) Unsupervised analysis of the five cellular populations separated by strain and infection. (C) Total number and phenotype of CD4+ T cells according to expression intensity of IL-4, IL-10, IL-17, TNF, and IFN-γ. (D) Total number and phenotype of CD8+ T cells according to expression intensity of IL-4, IL-10, IL-17, TNF, and IFN-γ. Statistical analyses were conducted between each strain and its uninfected group and between the two strains at the same infection time. *N* = 5–6 biological replicates per group. Results are represented by the mean ± SD *p < 0.05, **p < 0.01, ***p < 0.001, ****p < 0.0001. Data were analyzed using one-way ANOVA with Tukey post hoc testing. WT: wild-type.

The unsupervised t-SNE analysis revealed four main immune cell populations in the heart: lymphocytes, monocytes, heart macrophages (HMs), and neutrophils (Figure 4A). When stratified by group (Figure 4B), we observed an expansion of monocytes in infected animals, mainly in KO mice, whereas neutrophils and HMs displayed only subtle alterations. Interestingly, lymphocytes formed heterogeneous clusters, even in naive animals, suggesting that cardiac lymphocyte populations display intrinsic phenotypic diversity that is further shaped during infection.

We first focused on T cell subsets (Figure 4C,D). Infected ST2^–/–^ mice exhibited a reduced number of CD4+ T cells compared to their naive counterparts; however, this was likely attributable to an elevated baseline population of these cells in the hearts of naive ST2^–/–^ mice (Figure 4C). Phenotypic analysis revealed that CD4+ T cells expressed IL-10 during infection across groups, but only ST2^–/–^ infected mice showed higher expression intensity, measured as the mean fluorescence intensity (MFI) of TNF-α and IFN-γ, indicating a shift toward a proinflammatory phenotype. The predominance of IFN-γ in ST2^–/–^ infected CD4+ T cells was further confirmed by cytokine ratios, where IFN-γ expression outweighed that of IL-10, IL-4, and IL-17 (Figure S4). Despite a reduced absolute number of Tregs in ST2^–/–^ infected mice (likely due to their altered CD4+ T cell pool in naive KO mice), their phenotype remained unchanged, in agreement with the low infiltration of Tregs typically seen in cardiac tissue^21^ (Figure S5).

Furthermore, we observed a robust infiltration of CD8+ T cells in infected mice, consistent with observations in heart tissue from Chagasic patients (Figure 4D). These CD8+ cells displayed decreased IL-4 and TNF-α expression during infection, but an increased level of production of IL-10. Notably, only ST2^–/–^ infected mice displayed strong IFN-γ expression, with cytokine ratio analysis confirming IFN-γ dominance over IL-10 and IL-17 (Figure S6). These findings highlight the critical role of the IL-33/ST2 axis in modulating T cell response during infection, with its absence predisposing it to an IFN-γ-driven inflammatory profile.

### 2.5 ST2 Signaling Modulates γδ T Cell Effector Function But Not Recruitment during Cardiac Infection with *T. cruzi*

Our analysis of conventional T cells revealed a potent IFN-γ-driven response, particularly in ST2^–/–^ hearts (Figure 4). However, the heterogeneity of the lymphocyte clusters in our initial t-SNE analysis suggested that other key players were contributing to the cardiac immune landscape. This led us to hypothesize a role for γδ T cells, a lymphocyte population known for its rapid response to cellular stress and its established importance in human CD peripheral response.^25,26^ Human Vγ9 Vδ2+ cells recognize *T. cruzi* phosphoantigens and serve as an important source of cytokines. In mice, however, this subset is absent; instead, their γδ T cells respond to damage-associated molecular patterns.^27^ On this basis, we hypothesized that γδ T cells are recruited to the heart in response to infection and may contribute to the local inflammatory milieu, potentially through sensing IL-33/ST2-dependent signals.

To specifically investigate this population, we refined our unsupervised analysis to visualize the expression of key markers (Figure 5A). This approach confirmed that while CD4+ and CD8+ T cells formed their own distinct clusters, a significant and separate population of lymphocytes expressed the γδ T cell receptor, forming a unique island that clearly expanded upon infection. This demonstrates that γδ T cells represent a substantial component of the cardiac T cell infiltrate during *T. cruzi* infection.

**Figure 5:**
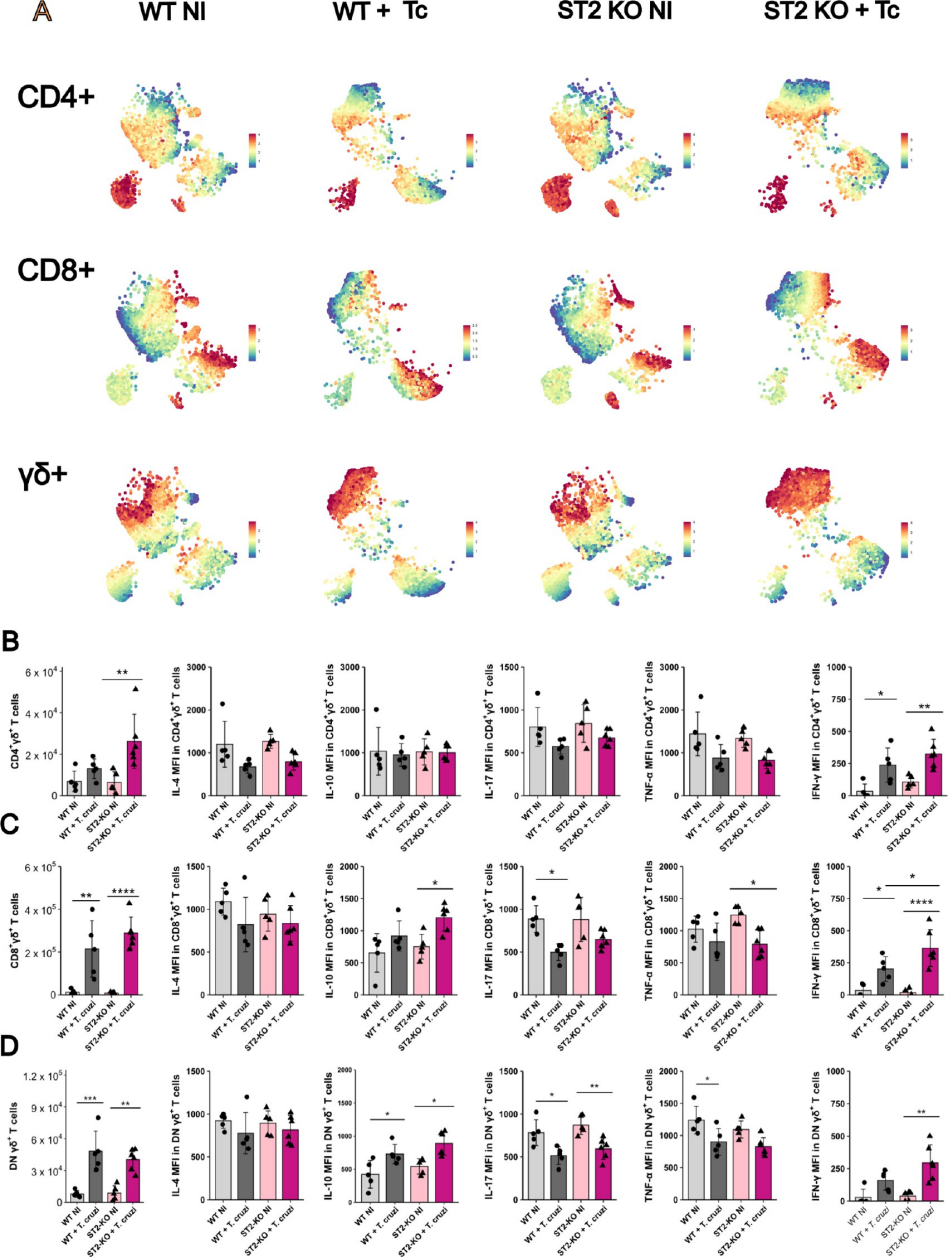
Analysis of lymphocyte clusters and γδ T cells phenotypes by flow cytometry in the heart of WT and ST2^–/–^ mice during *T. cruzi* infection at 20 dpi. (A) t-SNE feature plots showing expression intensity of CD4, CD8, and the γδ T cell receptor, revealing a distinct and expanding γδ T cell population. (B–D) Absolute numbers and MFI of key cytokines for cardiac γδ T cell subsets: (B) CD4+γδ+ T cells, (C) CD8+γδ+ T cells, and (D) double-negative (CD4–CD8−) γδ+ T cells. Statistical analyses were conducted between each strain and its uninfected group and between the two strains at the same infection time. *N* = 5–6 biological replicates per group. Results are represented by the mean ± SD *p < 0.05, **p < 0.01, ***p < 0.001, ****p < 0.0001. Data were analyzed using one-way ANOVA with Tukey post hoc testing. WT: wild-type.

We next quantified and phenotyped the cardiac γδ T cell compartment, stratifying it into CD4+, CD8+, and double-negative (DN) subsets. Phenotypic analysis confirmed the recruitment of multiple γδ T cell subsets to the heart following infection (Figure 5B–D). Phenotypically, these cells adopted a potent effector profile. In particular, the expression intensity of IFN-γ was significantly upregulated upon infection across all γδ T cell subsets.

The absolute numbers of infiltrating γδ T cells did not differ between infected WT and ST2^–/–^ mice, indicating that the primary signals driving their recruitment to the heart are ST2-independent. However, the ST2 pathway appears to modulate their effector function. For the CD8+γδ+ subset, the MFI of IFN-γ was significantly higher in infected ST2^–/–^ mice compared to their WT counterparts, and when compared to naive mice, IFN-γ expression in the DN γδ+ subset was higher only for KO mice (Figure 5B,C). This suggests that ST2 signaling normally acts to modulate the intensity of the IFN-γ response in these cells. Despite this response, the fundamental polarization toward a pro-inflammatory phenotype was an ST2-independent event, as the ratios of IFN-γ relative to IL-4, IL-10, and IL-17 were similarly and significantly elevated in both infected groups compared to naive controls (Figure S7).

Together, these results identify γδ T cells as a major source of IFN-γ in the *T. cruzi*-infected heart. Although their recruitment occurs independently of ST2, deficiency in this signaling pathway enhances IFN-γ expression intensity in some γδ T cell subsets.

### 2.6 ST2 Deficiency Reshapes Cardiac Macrophage Subsets toward a Pro-Inflammatory Profile during *T. cruzi* Infection

Beyond the T cell compartment, we next investigated the myeloid response. Cardiac heart macrophages (HM) were stratified into four major subsets based on MHC-II and CCR2 expression.^28^ Dimensional reduction analysis revealed these canonical populations but also highlighted the existence of two distinct MHC-II+CCR2+ clusters, differentiated by their CX3CR1 expression (populations A and B) (Figure 6A,B). Notably, MHC-II+CCR2+CX3CR1^high^ HMs emerged as the main cytokine-producing population, displaying a multifunctional profile (Figure S8).

**Figure 6:**
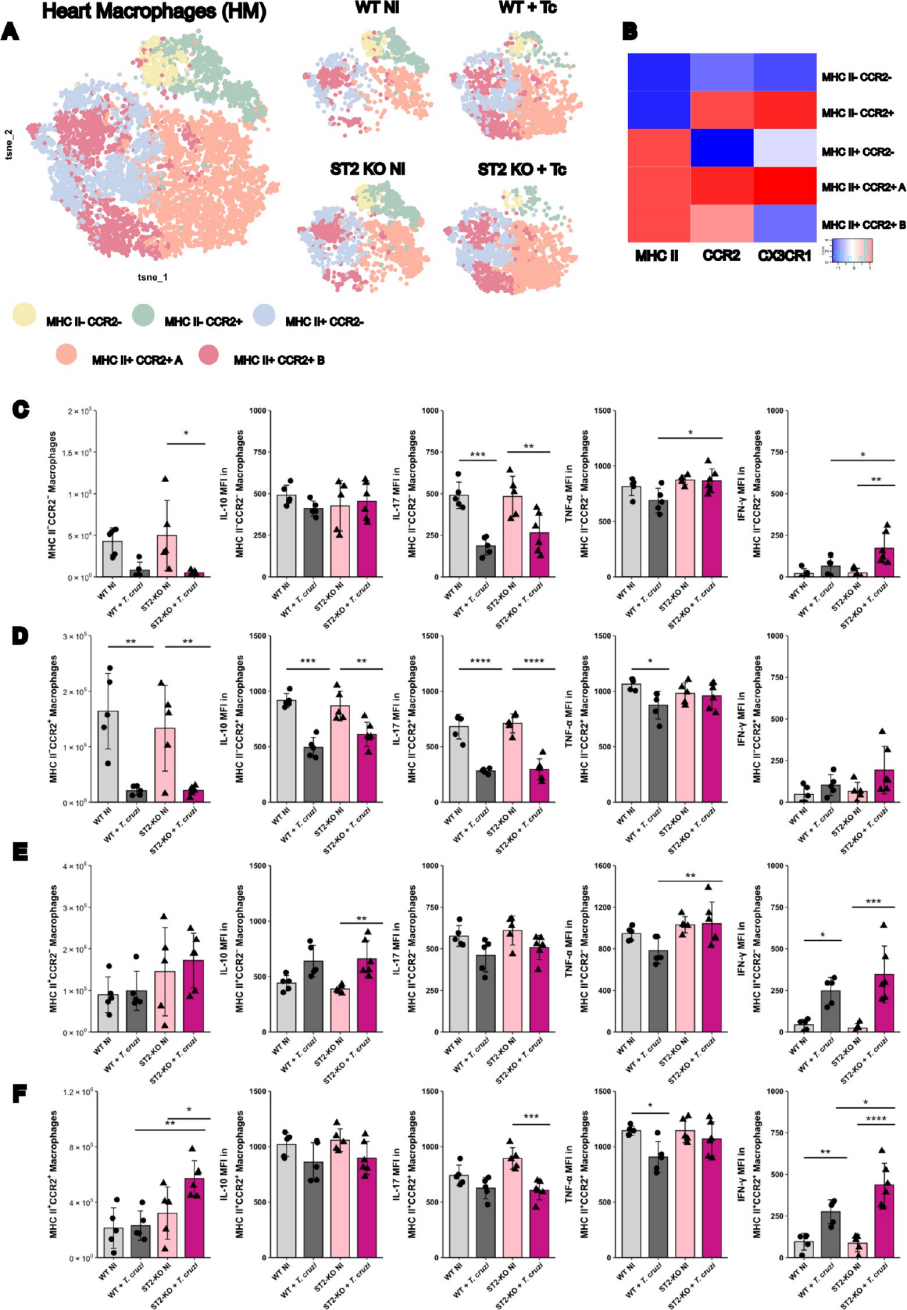
Characterization of heart macrophage clusters by flow cytometry in the heart of WT and ST2^–/–^mice during *T. cruzi* infection at 20 dpi. (A) t-SNE plots of heart macrophages (HM), concatenated for all groups (left), or separated by experimental group (right). Colors represent distinct HM clusters. (B) Heatmap showing the median expression intensity of MHC II, CCR2, and CX3CR1 used to define the five HM clusters identified in (A). (C) Total number and phenotype of MHC II–CCR2–HMs according to expression intensity of IL-10, IL-17, TNF, and IFN-γ. (D) Total number and phenotype of MHC II– CCR2+ HMs according to expression intensity of IL-10, IL-17, TNF, and IFN-γ. (E) Total number and phenotype of MHC II+ CCR2–HMs according to expression intensity of IL-10, IL-17, TNF, and IFN-γ. (F) Total number and phenotype of MHC II+ CCR2+ HMs according to expression intensity of IL-10, IL-17, TNF, and IFN-γ. Statistical analyses were conducted between each strain and its uninfected group and between the two strains at the same infection time. *N* = 5–6 biological replicates per group. Results are represented by the mean ± SD *p < 0.05, **p < 0.01, ***p < 0.001, ****p < 0.0001. Data were analyzed using one-way ANOVA with Tukey post hoc testing. WT: wild-type.

Infection disrupted the balance between macrophage subsets in both WT and ST2^–/–^ mice, with an expansion of MHC-II+CCR2+ macrophages (Figure 6A). However, in KO animals, this shift was accompanied by a marked reduction of double-negative (MHC-II–CCR2−) and MHC-II–CCR2+ populations (Figure 6C,D).

Phenotypically, double-negative macrophages, typically considered tissue-resident and regulatory, exhibited unexpected pro-inflammatory features in ST2^–/–^ infected mice, including higher TNF and IFN-γ expression compared to WT-infected mice (Figure 6C). This profile was further reflected in an elevated IFN-γ/IL-10 ratio within this subset (Figure S9). MHC-II–CCR2+ macrophages decreased in numbers during infection, with reduced IL-10 and IL-17 expression, consistent with a loss of regulatory potential (Figure 6D). In contrast, MHC-II+CCR2– macrophages remained numerically stable, but ST2^–/–^ mice displayed enhanced IL-10 and TNF expression, and elevated IFN-γ compared to controls (Figure 6E).

The most notable change was observed in the MHC-II+CCR2+ subset, which expanded significantly only in ST2^–/–^ mice during infection (Figure 6F). This subset, typically associated with monocyte-derived inflammatory macrophages, showed reduced levels of IL-17 in KO mice, but increased levels of IFN-γ production relative to those in WT controls. Together with the heatmap data (Figure S8), these findings highlight MHC-II+CCR2+ macrophages as a major pro-inflammatory source within the infected heart, particularly in the absence of ST2 signaling.

Collectively, these results reveal a robust modulation of cardiac macrophage compartments in ST2-deficient mice, characterized by the emergence of pro-inflammatory features in typically regulatory double-negative macrophages, coupled with the expansion of pathogenic double-positive subsets. This accumulation of pro-inflammatory, monocyte-derived macrophages is a hallmark of cardiac dysfunction and likely synergizes with the IFN-γ-skewed T cell response to drive the severe pathology observed in ST2^–/–^ mice.

### 2.7 Loss of IL-33/ST2 Signaling Skews Cardiac Monocyte and Neutrophil Populations toward an IFN-γ-Dominated Phenotype during *T. cruzi* Infection

Given the observed expansion of the MHC-II+CCR2+ HMs, we next investigated monocyte (MO) dynamics during infection. We stratified infiltrating MOs into Ly6C^high^ (classical, pro-inflammatory) and Ly6C^low^ (patrolling) subsets.

Flow cytometry analysis revealed that ST2^–/–^ infected mice displayed significantly increased cardiac infiltration of both Ly6C^high^ and, more prominently, Ly6C^low^ MOs compared to both infected WT mice and noninfected ST2^–/–^ controls (Figure 7B,C). Phenotypic profiling showed that infection reduced IL-4 expression in both MO subsets, while Ly6C^low^ MOs from ST2^–/–^ mice also exhibited decreased IL-17 levels. Interestingly, WT-infected mice displayed a decrease in TNF compared to their naive counterparts. In contrast, infection was associated with increased levels of IFN-γ and MHC-II expression across both MO subsets. Accordingly, cytokine ratio analysis evidenced a proinflammatory polarization, as Ly6C^high^ MOs from ST2^–/–^ mice displayed significantly higher IFN-γ/IL-4 and IFN-γ/IL-10 ratios than those from WT-infected mice (Figure S7).

**Figure 7:**
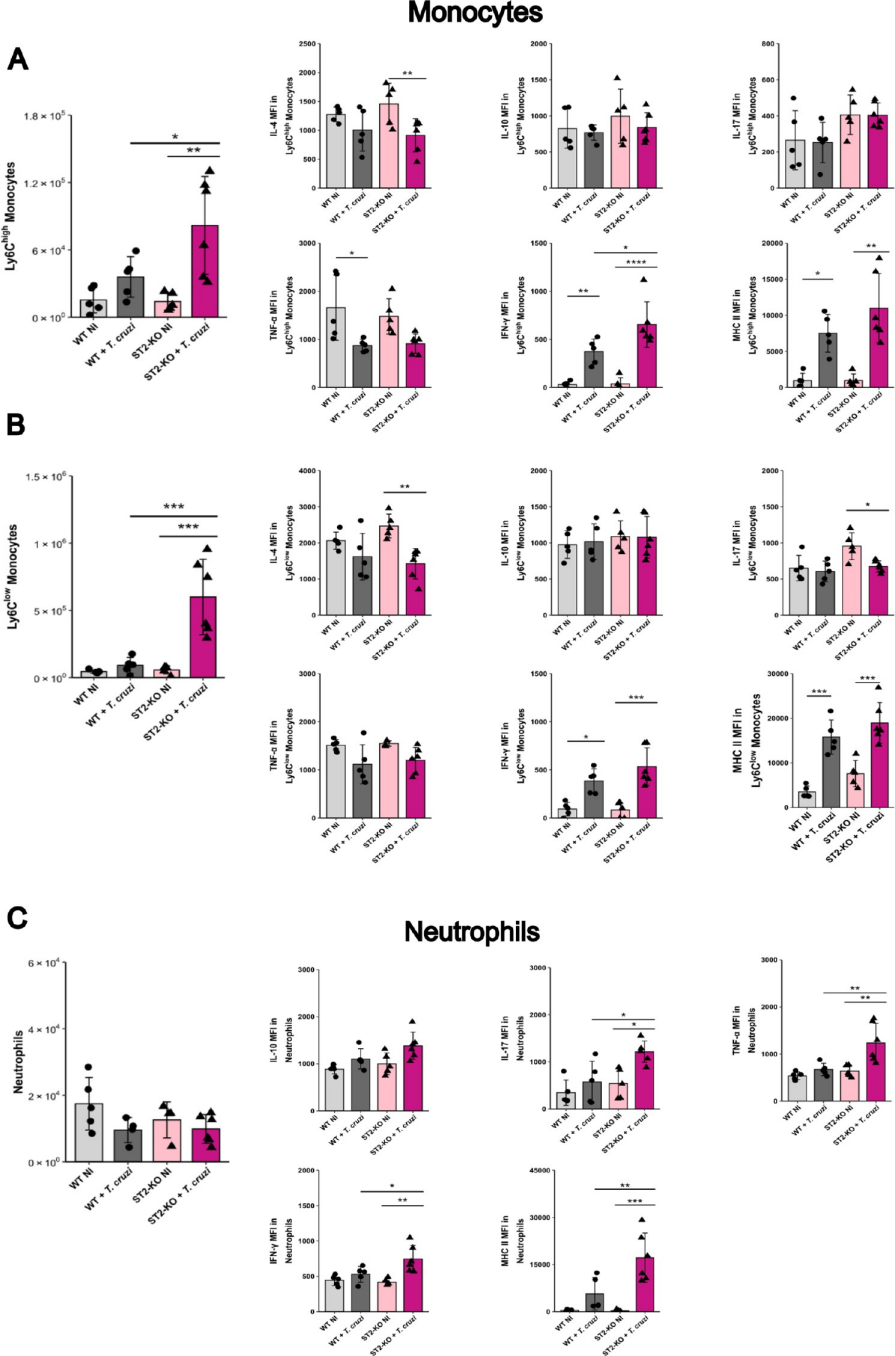
Profiling of monocyte subsets and neutrophils by flow cytometry in the heart of WT and ST2^–/–^ mice during *T. cruzi* infection at 20 dpi. (A,B) Total number and phenotypic profiling of Ly6C^high^ (A) and Ly6C^low^, (B) monocytes based on the expression of IL-4, IL-10, IL-17, TNF, IFN-γ, and MHC-II. (C) Total number and phenotypic profiling of neutrophils based on the expression of IL-10, IL-17, TNF, IFN-γ, and MHC-II. Statistical comparisons were performed between each infected group and its uninfected control, and between WT and ST2^–/–^ mice at the same infection time point. *N* = 5–6 biological replicates per group. Data are presented as mean ± SD *p < 0.05; **p < 0.01; ***p < 0.001; ****p < 0.0001 (one-way ANOVA with Tukey’s post hoc test). WT: wild-type.

In addition to MOs, we examined the cardiac neutrophil (NEU) population. As evidenced by the absence of statistical significance, no difference was observed in the total number of neutrophils infiltrating the heart tissue among any of the evaluated groups (Figure 7D). However, NEUs from ST2^–/–^ infected mice acquired a distinct phenotype, with markedly increased expression of IL-17, IFN-γ, TNF, and MHC II compared to that of both naive and WT-infected controls.

Taken together, these findings indicate that the loss of the ST2 receptor drives an imbalance in both MOs and NEUs responses during *T. cruzi* infection, favoring enhanced recruitment of MO subsets. Since Ly6C^high^ MOs are closely linked to cardiac inflammation and dysfunction,^29^ and NEUs have been linked with infectious myocarditis,^28^ these alterations may also contribute to heart damage and electrical abnormality development.

### 2.8 ST2 Deficiency Disrupts Intraventricular Conduction during Experimental *T. cruzi* Infection in Mice

To evaluate whether the structural abnormalities and exacerbated inflammation driven by IFN-γ-responses observed in ST2^–/–^ mice translated into altered electrical activity, we performed electrocardiography (EKG) at 20 dpi, a time point coinciding with peak myocarditis and elevated CK-MB levels (Figure 8). The EKG records the heart’s electrical activity as a waveform composed of characteristic intervals. The PR segment reflects atrioventricular (AV) conduction through the AV node; the QRS complex represents ventricular depolarization; the QRS axis provides information about the direction of ventricular activation; and the QT interval measures the time from ventricular depolarization to repolarization, serving as a marker of arrhythmia risk.

**Figure 8:**
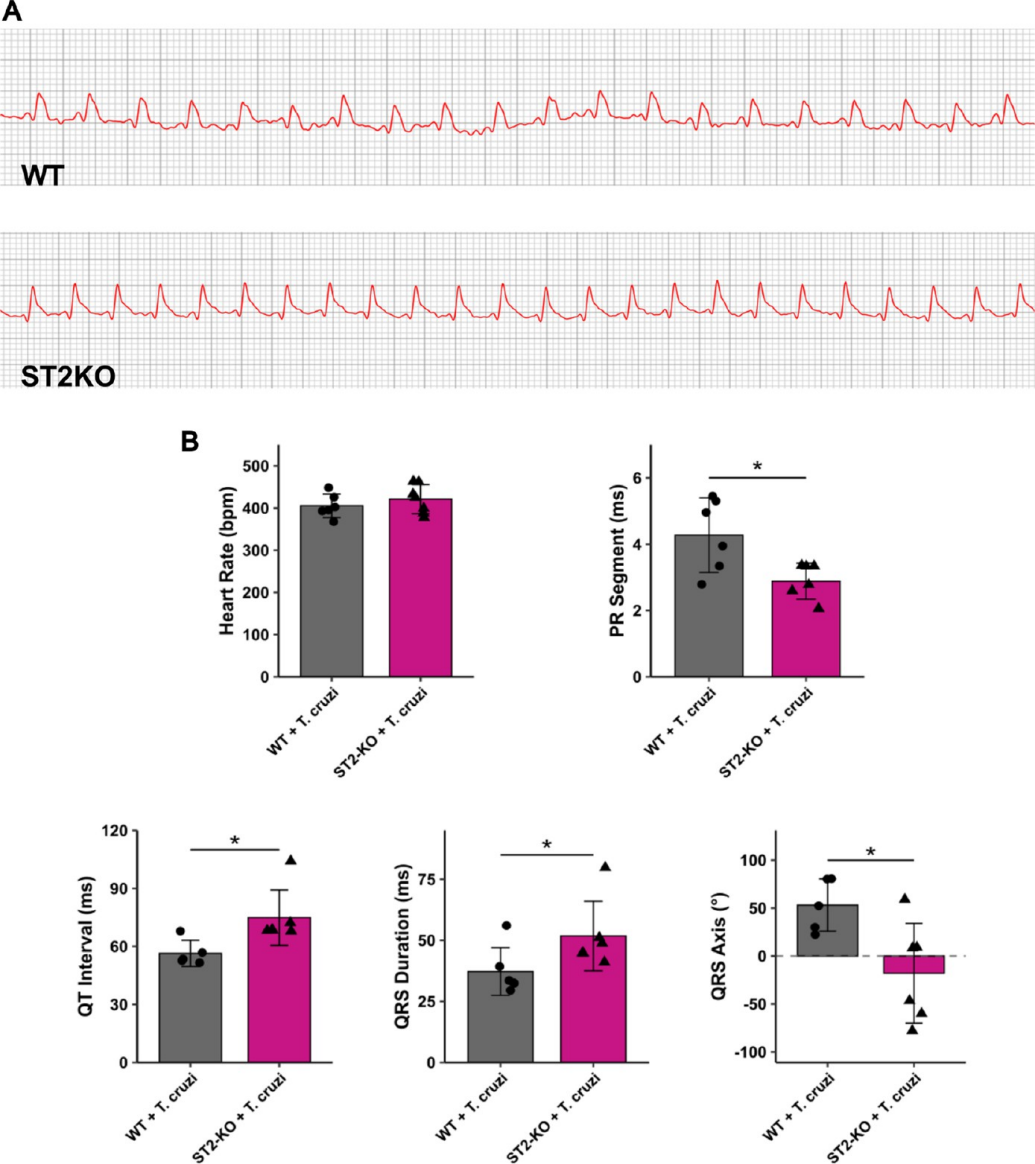
ST2 deficiency disrupts intraventricular conduction during *T. cruzi* infection at 20 dpi. (A) Representative EKG tracings from lead II (DII), recorded at 50 mm/s. Note the wider QRS complex in the ST2^–/–^ tracing. (B) Quantification of key electrocardiographic parameters: heart rate (bpm), PR segment (ms), QT interval (ms), QRS duration (ms), and QRS axis (°). Data are from *N* = 5–6 mice per group. Results are shown as mean ± SD. Statistical analysis was performed using the *t*-test. *p < 0.05.

A review of the EKG tracings showed that KO mice exhibited suggestive abnormalities, including a prolonged QRS complex (Figure 8A). Heart rate did not differ between groups, indicating that conduction abnormalities were not secondary to chronotropic changes (Figure 8B). However, ST2^–/–^ mice exhibited a reduction in the PR interval compared with WT animals, suggesting accelerated AV nodal conduction. In contrast, intraventricular conduction was impaired: ST2^–/–^ mice showed significantly prolonged QRS duration together with a marked leftward deviation of the QRS axis, consistent with an intraventricular conduction block (Figure 8A,B). Finally, QT intervals were significantly increased in ST2^–/–^ mice, indicating delayed ventricular repolarization and a pro-arrhythmic electrical substrate (Figure 8B).

In summary, the absence of ST2 signaling drives a unique electrical remodeling profile. While conduction through the AV node is faster, the subsequent spread of the electrical impulse through the ventricles is significantly impaired, suggesting a phenotype of intraventricular conduction disease. Measured EKG parameters are shown in Table S2.

### 2.9 Multivariate Analysis Reveals ST2 as a Central Regulator of Immunopathology Balance in *T. cruzi* Infection

To better understand how ST2 deficiency shapes the overall disease pattern, we performed a multifactorial analysis integrating cardiac immune cell populations, serum CK-MB (as a marker of myocardial damage), and tissue fibrosis. The correlogram (Figure 9A) revealed a strong inflammatory signature associated with cardiac injury, with robust positive correlations linking CK-MB levels, fibrosis area, CD8+ T cells, and monocytes, particularly those with a Ly6C^low^ phenotype.

**Figure 9:**
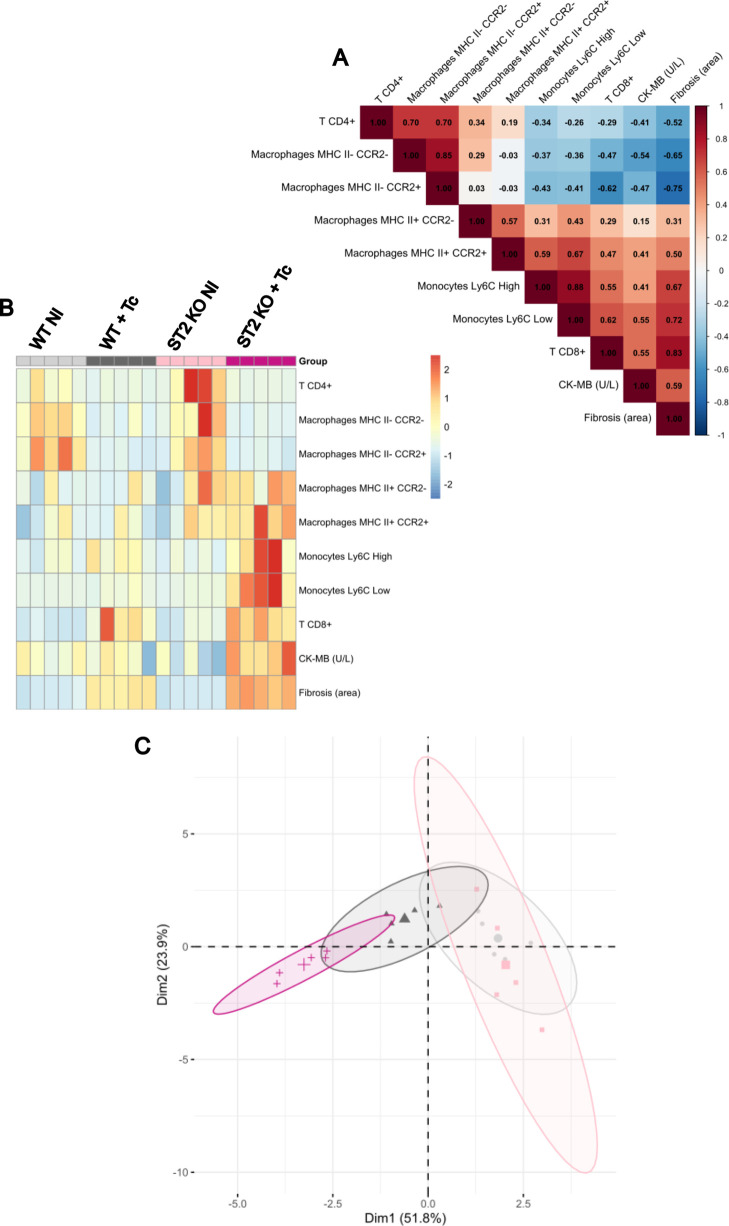
Multivariate analysis reveals exacerbated inflammatory and pathological signatures in ST2-deficient mice during *T. cruzi* infection at 20 dpi. (A) Spearman’s correlogram showing correlations among cardiac immune populations, CK-MB, and fibrosis. Color intensity and square size indicate correlation strength (red = positive, blue = negative). (B) Heatmap of analyzed variables in WT and ST2^–/–^ mice, uninfected (NI), or acutely infected with *T. cruzi* (Tc). Red colors represent values above the mean; blue colors represent values below the mean. (C) Principal component analysis (PCA) integrating all variables. Each symbol represents an individual mouse (circles: WT NI; squares: ST2^–/–^ NI; triangles: WT-infected; + symbol: ST2^–/–^ infected); ellipses denote 95% confidence intervals for each group. Axes represent the first two principal components (Dim1 and Dim2).

This relationship was further illustrated in the heatmap (Figure 9B), where infected ST2^–/–^ mice displayed markedly higher values for CD8+ T cells, monocytes, CK-MB, and fibrosis compared with WT mice. These patterns highlight the exacerbated inflammatory response and tissue damage driven by loss of ST2 signaling.

Principal component analysis (PCA) consolidated these observations (Figure 9C). Both uninfected groups (WT NI and ST2^–/–^ NI) clustered tightly together along Dim1, reflecting a shared basal homeostatic phenotype. Infection induced a pronounced shift in both the WT and ST2^–/–^ groups, consistent with the establishment of an inflammatory profile. Notably, the infected ST2^–/–^ group formed its own distinct cluster, positioned significantly further along Dim1 than the infected WT group. This greater deviation from the homeostatic state signifies a more extreme and severe immunopathological profile in the absence of ST2 signaling.

Taken together, this integrated analysis consolidates our findings and demonstrates that the IL-33/ST2 axis is a critical regulator of cardiac immunopathology during *T. cruzi* infection. Its absence results in unrestrained inflammatory responses that drive heightened myocardial injury, and fibrosis, culminating in more severe cardiomyopathy.

## 3 Discussion

CD is a neglected infection affecting millions globally, characterized by lasting morbidity and progressive tissue dysfunction largely driven by host inflammatory responses.^1^ Disease outcome is tightly linked to the balance between protective and pathogenic immune responses during *T. cruzi* infection.^10,30^ In particular, Th1-driven IFN-γ responses, while necessary for parasite control, have been consistently implicated in the development of myocarditis and progression to chronic cardiomyopathy.^31,32^ Conversely, regulatory responses have been associated with attenuated inflammation and a more favorable clinical trajectory.^33,34^

In this context, the IL-33/ST2 axis emerges as a central immunomodulatory pathway. IL-33, an alarmin cytokine released upon tissue damage, signals through its receptor ST2 to exert pleiotropic effects that can be either protective or pathological.^16^ In the heart, IL-33 has been shown to limit myocarditis by dampening Th1 responses and promoting IL-4 production by macrophages, and T cells.^35^ In helminth infections, dysregulation of this axis has been associated with uncontrolled Th1/Th17 activity and impaired regulatory T cell responses.^36,37^ Yet, its role in shaping the cardiac immunopathology of *T. cruzi* infection has remained unresolved. Our study aimed to fill this gap by characterizing the course of infection in ST2-deficient mice.

We observed that ST2^–/–^ mice exhibited an initial reduction in parasitemia but levels converged with WT animals by 9 dpi. This transient phenotype suggests that IL-33/ST2 signaling is dispensable for sustained systemic parasite control. A similar pattern has been reported in *Plasmodium berghei* malaria, where IL-33/ST2 deficiency did not affect parasitemia, but is crucial in regulating pathology.^38,39^ Importantly, the absence of parasitemia differences does not preclude altered parasite burden in tissues, as described for IL-17 in *Trypanosoma brucei* infection.^40^ Notably, the accelerated mortality observed in ST2^–/–^ mice points to immunopathological drivers rather than uncontrolled parasite replication. Myocarditis is a key determinant of morbidity and prognosis in CD.^41,42^ The absence of ST2 signaling led to early onset (7 dpi) and severe myocarditis, significantly elevated markers of cardiac damage (CK-MB), extensive, disorganized fibrosis, and a complex electrical conduction disease characterized by both accelerated atrioventricular and impaired intraventricular conduction.

To understand the cellular drivers of this severe cardiomyopathy, we evaluated the cardiac immune infiltrate. The dramatic systemic monocytosis observed at 15 dpi preceded a robust influx of monocytes into the heart at 20 dpi. In cardiac injury, Ly6C^high^ monocytes are typically pro-inflammatory, while Ly6C^low^ monocytes are associated with patrolling and tissue repair.^43^ In general, innate lymphoid cells, natural killer (NK) cells, and T lymphocytes are the major producers of IFN-γ; however, myeloid cells may also be significant, early producers of this cytokine, particularly in the context of protozoan infections, and tissue injury. For instance, in experimental trypanosomatid infections, both monocytes and neutrophils have been identified as early sources of IFN-γ, helping to shape the initial Th1 response.^44,45^ Similarly, in sterile models of tissue damage, such as myocardial infarction, infiltrating monocytes, and macrophages are known to release a suite of inflammatory mediators that includes IFN-γ, directly contributing to the local inflammatory milieu.^46^ In our model, this functional reprogramming of monocytes likely fueled the expansion of pathogenic monocyte-derived macrophages, and reinforced an IFN-γ-dominated environment, exacerbating tissue damage and fibrosis.

Regarding the cardiac macrophage landscape, we observed two key pathogenic events in ST2^–/–^ mice: (1) the typically resident and regulatory MHC-II–CCR2– macrophages underwent a functional switch, adopting a pro-inflammatory profile by producing TNF-α, and IFN-γ; (2) the pathogenic, monocyte-derived MHC-II+CCR2+ population expanded. Our refined t-SNE analysis revealed that this expansion was dominated by a CX3CR1^low^ IFN-γ^high^ subset, a phenotype associated with freshly recruited, highly inflammatory macrophages known to drive cardiac dysfunction. Furthermore, the normally homeostatic MHC-II–CCR2+ macrophage population, which may have regulatory potential, was depleted during infection. This scenario, losing regulatory populations while gaining and activating pathogenic ones, differs from other models of infectious myocarditis, where an increase in almost all macrophage subsets can be observed.^29^ Neutrophils, although not increased in number, acquired a hyperactivated phenotype with elevated IL-17, IFN-γ, and MHC-II expression, paralleling reports that activated neutrophils exacerbate myocarditis in infectious and immune contexts.^47^ Together, these alterations suggest that IL-33/ST2 signaling normally restrains pro-inflammatory activation across multiple myeloid compartments.

A central finding of our study was that the IFN-γ-rich environment in ST2^–/–^ hearts failed to improve parasite clearance. Instead, these mice displayed a higher cardiac parasite burden. (We note that this quantification was performed by absolute qPCR normalized to total DNA input; while this method is validated for comparative analysis, we acknowledge the absence of a host reference gene as a study limitation). This result highlights that the efficacy of IFN-γ is both context- and cell-dependent and relies on other effector mechanisms to achieve parasite control.^48,49^ Cardiomyocytes are targets of *T. cruzi* infection and active sources of chemokines and cytokines, which together induce iNOS and nitric oxide (NO) production, leading to potent trypanocidal activity.^50^ Indeed, the stimulation of infected cardiomyocytes with IFN-γ induces robust NO synthesis, and restricts parasite growth. Therefore, functional cardiomyocytes act as both producers and responders to key cytokines, amplifying the local antiparasitic response. In this context, the cardiomyocyte degeneration we observed in ST2^–/–^ mice likely impaired the ability of these cells to respond to IFN-γ and, consequently, to produce essential effector molecules, creating a permissive environment that facilitated parasite replication. This hypothesis is supported by our data showing lower levels of NO in ST2^–/–^ mice despite robust IFN-γ production. Nitric oxide production (via iNOS) in response to IFN-γ is typically optimized by costimulatory signals that converge on the MyD88 signaling cascade.^51^ The IL-33/ST2 pathway signals through MyD88 and MAP kinases (ERK1/2, p38, and JNK), leading to NF-κB activation and transcriptional induction of inflammatory mediators, including iNOS.^16,17^ Therefore, the disruption of this pathway may impair MyD88-dependent NO synthesis. In *T. cruzi* infection, MyD88-deficient cardiomyocytes exhibit reduced iNOS expression as infection progresses, despite comparable parasite burdens.^52^ Without sufficient NO, IFN-γ signaling becomes ineffective at killing intracellular parasites, acting primarily as a driver of host cell apoptosis and bystander tissue damage.^53,54^ Furthermore, insights from other intracellular parasites, such as *Cryptosporidium*, reveal that IFN-γ acts most effectively on uninfected bystander cells, “priming” them to resist future invasion and thereby limiting pathogen spread.^55^ The widespread cardiomyocyte degeneration in ST2^–/–^ hearts would likely disrupt this protective network, further compromising tissue resistance to infection. Damaged or degenerating cells would not only fail to produce their own mediators but could also become hyporesponsive to IFN-γ signals from the microenvironment, preventing the priming of adjacent tissue and facilitating the infection of neighboring cells.

Additionally, parasite control is not solely dependent on IFN-γ and NO. The cytotoxic activity of CD8+ T cells and NK cells, mediated by granzymes and perforins, is another essential mechanism of intracellular parasite control. The IL-33/ST2 axis has been shown to play a crucial role in enhancing the cytotoxic potential of these cells.^56,57^ Although we did not measure these molecules, it is plausible that in ST2^–/–^ mice, this cytotoxic pathway is also impaired. This mechanism likely explains the disorganized parasite nests observed amidst cardiomyocyte debris: infected cells succumb to damage, releasing parasites into an inflammatory niche where they infect neighboring cells. These bystander cells, already compromised by the early inflammation, are likely unable to mount an effective IFN-γ-mediated antiparasitic response. This cycle could underlie the increased chronic parasitism observed in ST2^–/–^ hearts.

The IFN-γ-rich milieu also likely contributed directly to the severe and disorganized fibrosis observed. While inflammation is a known precursor to fibrosis, IFN-γ can play a direct, pathological role in tissue remodeling. Recent studies in models of chronic viral lung injury have shown that IFN-γ released by tissue-resident CD8+ T cells acts on macrophages to drive chronic IL-1β production, which in turn impairs alveolar regeneration and pulmonary function and promotes fibrosis.^58,59^ A similar IFN-γ-macrophage axis may operate in ST2^–/–^ hearts, linking inflammation to pathological tissue remodeling and conduction system dysfunction.

Another nuanced finding was the selective decrease in the level of TNF-α expression in some infected cell populations, despite the inflamed milieu. While this could reflect a kinetic effect, with the 20 dpi time point being past the already described precocious TNF-α peak in myocarditis,^60^ a more elegant mechanism has recently been proposed. Severe cardiac stress and inflammation can trigger a powerful compensatory neuroimmune reflex via the vagus nerve, which acts to suppress TNF-α production to limit further damage.^61^ Notably, *T. cruzi* infection can cause cardiac denervation and neuronal loss, potentially impairing this brain–heart regulatory axis. Evaluating neuroimmune reflex integrity during *T. cruzi* infection could provide key insights into the failure to restrain inflammation in Chagas cardiomyopathy.

Furthermore, our investigation into γδ T cells was prompted by their established role in human CD. In infected individuals, circulating γδ T cells, particularly the double negative (DN) subset, are potent cytokine producers whose functional profiles critically diverge with disease severity. Individuals with the asymptomatic, indeterminate form tend to display a more balanced, self-regulating γδ T cell phenotype, sometimes coexpressing IFN-γ and IL-10, correlating with preserved ventricular function. In contrast, patients who develop severe cardiomyopathy exhibit a highly pro-inflammatory, IFN-γ-dominant response from these cells, which is strongly associated with worse cardiac function.^26^ This activation in humans is often driven by nonpeptidic parasite components, such as glycoconjugates.^25^ However, the dynamics of these cells within the heart during experimental murine infection have remained largely unexplored, partly due to key biological differences, as murine γδ T cells primarily respond to host-derived damage signals rather than the parasite-derived phosphoantigens recognized by human Vγ9 Vδ2 T cells.

Here, we demonstrate a substantial recruitment of multiple γδ T cell subsets (CD4+, CD8+, and DN) into cardiac tissue during acute infection. These infiltrating cells were potent producers of IFN-γ, mirroring the pro-inflammatory signature observed in human patients with severe cardiomyopathy. The recruitment of γδ T cells to the heart was ST2-independent, suggesting that other damage-associated signals released during infection are sufficient to attract this sentinel population. Patients with indeterminate disease retain a balanced DN γδ phenotype, coexpressing IFN-γ and IL-10, while those with cardiomyopathy exhibit an IFN-γ-dominant profile associated with worse cardiac function. Notably, *T. cruzi*-derived glycoconjugates activate human DN γδ T cells, skewing them toward IFN-γ in cardiac patients but toward IL-10 in indeterminate individuals.^26^ In our mouse model, for DN γδ T cells, IFN-γ production was significantly increased only in ST2^–/–^ mice, while IL-10 production was upregulated in both infected groups. This suggests that IL-33/ST2 signaling may be particularly important for maintaining the regulatory phenotype of this specific subset. In its absence, these cells shift from a balanced IL-10^+^/IFN-γ^+^ state toward a more pathogenic IFN-γ^high^ profile, similar to what is observed in human patients with severe cardiac disease.

The functional consequence of this inflammation, fibrosis, and structural damage was a complex electrical remodeling. Alterations included prolonged PR intervals, and a higher incidence of atrioventricular conduction defects, consistent with sinoatrial node and conduction system inflammation.^62,63^ This is a clinically relevant finding, as the disorganized fibrosis we observed could create scar tissue that impedes normal electrical flow, forming a substrate for ventricular arrhythmias, which likely contributes to the accelerated mortality in these mice.

Our study provides a compelling mechanistic counterpart to the recent work by Boccardo and colleagues,^21^ which showed that early IL-33 supplementation protects against skeletal muscle damage by expanding tissue-repair regulatory T cells (trTregs) and Type 2 innate lymphoid cells (ILCs2). Here, we show that the genetic ablation of the ST2 receptor unleashes the Th1/IFN-γ response known to suppress these trTreg and ILC2 populations.^64,65^ The IFN-γ-dominated environment in our ST2^–/–^ mice likely creates a nonpermissive niche for these reparative cells, leading to unchecked tissue destruction.

Multivariate analyses integrating immune infiltrates, fibrosis, and CK-MB levels revealed that ST2^–/–^ mice clustered distinctly from WT-infected animals, with CD8+ T cells, monocytes, and fibrosis emerging as key drivers of separation. These data place IL-33/ST2 as a central regulatory player maintaining the balance between protective immunity and pathological inflammation. The severe acute pathology observed in ST2^–/–^ mice has profound implications for the chronic phase of the disease. The increased parasite persistence, extensive and disorganized fibrosis, and remaining areas of tissue degeneration at 100 dpi establish a foundation for more severe chronic Chagasic cardiomyopathy.

## 4 Conclusion

Our study highlights the IL-33/ST2 signaling axis as a crucial regulator of cardiac homeostasis during *T. cruzi* infection. Its absence leads to a failure of immune response balance, with IFN-γ-driven inflammation and tissue damage that culminates in a more severe cardiomyopathy and heart dysfunction. These findings indicate that the ST2 receptor is a potential therapeutic target for modulating the immune response to prevent the progression of CD.

## 5 Materials and Methods

### 5.1 Ethics Statement

All animal procedures were conducted in strict accordance with the guidelines of the Brazilian College of Animal Experimentation (COBEA). The experimental protocols were reviewed and approved by the Ethics Committee on Animal Use (CEUA) of the Federal University of Minas Gerais (UFMG, Brazil) under protocol number 12/2024. All efforts were made to minimize animal suffering.

### 5.2 Mice

Female wild-type (WT) and ST2-deficient (ST2^–/–^) BALB/c mice, aged 8–10 weeks, were used for all experiments. WT BALB/c mice were sourced from the Central Animal Facility of UFMG. ST2^–/–^ mice on a BALB/c background were originally provided by Dr. João Santana da Silva (University of São Paulo, USP) and were bred and maintained at the animal facility for infected animals within the Department of Parasitology (ICB, UFMG). All mice were housed under a 12 h light/dark cycle with ad libitum access to standard chow (Presence, Primor, Brazil) and water.

### 5.3 *T. cruzi* Experimental Infection

Parasites of the *T. cruzi* Y strain (kept in Swiss mice) were used in all of the experiments. For infection assays, WT and ST2^–/–^ BALB/c mice received intraperitoneally inoculations of 1 × 10^3^ blood trypomastigotes suspended in sterile phosphate-buffered saline (PBS). Parasitemia was periodically monitored by counting motile trypomastigotes in 5 μL of fresh tail vein blood via light microscopy, as described by Brener (1962).^66^

### 5.4 Experimental Design

To investigate the contribution of the IL-33/ST2 pathway to experimental CD pathogenesis, WT and ST2^–/–^ mice were followed for up to 100 days postinfection to evaluate parasitemia dynamics, survival, and systemic immune profiles. Mice were euthanized at defined time points: day 0 (uninfected controls), days 7 and 20 (acute phase), and day 100 (chronic phase) postinfection (dpi). A total of 4–8 mice per group were used for each experimental time point. Euthanasia was induced with an overdose of xylazine (8.5 mg/kg) and ketamine (130 mg/kg). During necropsy, blood was collected by a cardiac puncture for biochemical assays. Hearts were promptly removed and sectioned longitudinally: half was snap-frozen for parasite burden determination, and the other half was fixed for histopathological analysis. For a separate cohort of animals at specific time points, hearts were collected, enzymatically digested, and processed for flow cytometry analysis, as described in Section 5.10.

### 5.5 Systemic Leukocyte Analysis

Total leukocyte counts in the tail vein blood were measured using a Bio-2900 Vet automated hematology analyzer. For differential counts, blood smears were prepared and stained with Panoptic fast stain (Laborclin, Brazil), and 100 leukocytes were identified and counted under a light microscope.

### 5.6 Histopathological and Morphometric Analysis

After removal and processing as described previously, heart fragments were fixed in a buffered 10% formaldehyde solution (Synth, Brazil) for 7 days. Subsequently, the samples were gradually dehydrated in ethanol, diaphanized in xylol, and included in paraffin blocks, obtaining 4 μm thick cuts with which histopathological slides were made and stained with hematoxylin and eosin (H&E) for histopathological, semiquantitative morphometric analysis. Cardiac lesions were analyzed by a blinded veterinary pathologist using a semiquantitative scoring system for myocarditis (Table 1).^23,67^ Briefly, the severity and extent of lesions were graded using a system from 0 to 4, where 0 = no inflammation; 1 = focal myocarditis; 2 = multifocal myocarditis; 3 = multifocal with extensive infiltration; and 4 = diffuse, widespread infiltration. To evaluate cardiac fibrosis, additional sections of hearts were stained with Masson’s trichrome and scanned at 200× magnification using an Axios Z.1 scanner (Zeiss). The ImageJ software version 1.54p (available from NIH, Bethesda, MD, USA) was used to quantify the fibrotic areas, as previously described.^24^

### 5.7 Tissue Parasite Burden Quantification

DNA was extracted from heart tissue (25 ± 2 mg of tissue) using a guanidine hydrochloride-based method. Briefly, cell lysis was obtained by a guanidine hydrochloride buffer. Sodium polyanetholesulfonate was removed by adding benzyl alcohol to the solution, and DNA was precipitated by sodium acetate in isopropanol solution. The pellet was washed in 70% ethanol and resuspended in 40 μL of Tris–EDTA buffer. Following resuspension, the DNA concentration and purity were determined using spectrophotometry (NanoDrop 2000c, Thermo Fisher). All samples were then normalized to a standard concentration to ensure a uniform input of 50 ng of DNA for each reaction. The final conditions in the PCR mixture were 1× Power SYBR Green ABI reagent (Thermo Fisher, USA), 2 μM of forward and reverse primers, and 50 ng of DNA, in a final volume of 10 μL per well. To quantify *T. cruzi* DNA, primers F (5′-3′ CGAGCTCTTGCCCACACGGGGCT) and R (5′-3′ CCTCCAAGCAGCGGATAGTTAGG) were used to amplify a 188 bp fragment from a satellite region gene.^68^ Samples were amplified in an ABI 7500 thermocycler (Applied Biosystems) with the following PCR conditions: predenaturation (10 min at 95 °C), 40 cycles of denaturation (15 s at 95 °C), and annealing (1 min at 60 °C). Analysis was performed using 7500 Software version 2.0.1 (Thermo Fisher, USA), and the results were determined using the standard curve method. Standard curves were constructed from serial dilutions of DNA extracted from 10^7^ *T. cruzi* trypomastigotes.

### 5.8 Biochemical Analyses

Serum was obtained from the whole blood by centrifugation at 5000 rpm for 10 min at room temperature. Creatine kinase-MB (CK-MB), a biomarker of cardiac injury, was quantified using a commercial colorimetric enzymatic kit (CK-MB UV K069, Bioclin Quibasa, Brazil) following the manufacturer’s protocol. Nitric oxide (NO) production was indirectly measured by quantifying nitrite levels in serum and heart tissue homogenates using the Griess reaction.^69^ Absorbance was read at 540 nm, and the nitrite concentration was calculated from a sodium nitrite standard curve. The data were obtained using an automated ELISA reader (Versa-Max/Molecular Devices Microplate Reader, USA) and Softmax Pro 5.3 software.

### 5.9 Electrocardiogram Recording

In vivo, noninvasive electrocardiography (EKG) was performed using an INcardio X electrocardiograph (Inpulse Animal Health, Florianópolis, SC, Brazil). Animals were anesthetized with 2.5% isoflurane and maintained at 1.5% (VetCase, Incotec, Serra, ES, Brazil). Mice were placed in a dorsal recumbency position on a wooden table covered with plastic material; electrocardiographic gel was applied, and four “alligator” electrodes were attached to the skin on the cranial and caudal limbs.^70^ Recordings were acquired for 5 min at a speed of 50 mm/s and *N* sensitivity (1 mV = 10 mm). Experiments were conducted in a quiet environment to reduce stress, and data were analyzed by using the INcardio X software.

### 5.10 Heart Digestion

Heart digestion was performed as described previously^71^ with modifications. Briefly, following euthanasia, mice were perfused with 3 mL of Hank’s Balanced Salt Solution (HBSS). Heart tissue (60 ± 2 mg) was placed in a 15 mL tube with 3 mL of HBSS plus brefeldin A (10 μg/mL) (Invitrogen). Digestion was performed for half an hour with 300 units of DNase I (Sigma-Aldrich), 625 units of collagenase II (Sigma-Aldrich), and 50 units of hyaluronidase II (Sigma-Aldrich). After digestion, the tubes were centrifuged at 250 *g* for 5 min at 4 °C, the supernatant was discarded, and the pellet was resuspended in 5 mL of ACK lysis buffer (Invitrogen) and incubated for 5 min at room temperature to remove red blood cells. Suspended cells were passed through a 70 μm filter and then prepared for flow cytometry.

### 5.11 Flow Cytometry

Flow cytometry was performed using a technique previously described.^71^ Cells were plated and incubated with brefeldin A (10 μg/mL; Invitrogen) for 4 h at 37 °C in the presence of 5% CO2. Then, 300 μL of PBS/BSA 1% (phosphate-buffered saline and 1% fetal bovine serum) was added and centrifuged at 250 *g* for 5 min at 4 °C. The supernatant was carefully removed, and PBS/BSA 1% Fc Block (Fc block, CD16/CD32) diluted 1/100 was added and incubated for 30 min.

Cell suspensions were labeled with fluorescently conjugated antibodies. Intracellular staining for cytokines and transcription factor Foxp3 was performed using the Foxp3/Transcription Factor Staining Buffer Set (eBioscience). Flow cytometry was performed on a FACSymphony A5 SE instrument (BD Biosciences), and analyses were performed using FlowJo version 10.10.0 (Becton Dickinson). All of the following Bioscience markers were used: CD4-BUV395 (clone GK1.5), CD8-BUV805 (clone 53–6.7) FOXP3-AF700 (clone MF-14), TNF-BV786 (clone MP6-XT22), IFN-γ-PECF (clone XMG1.2), IL-4-BV711 (clone 11B11), IL-10-BV421 (clone JES5), Viability-eFluor450 (65‑0863‑18), CD45-PECY (clone 30-F11), Ly6G-BUV563 (clone 1A8), CD11b-BB700 (clone M1/70), TCRγδ-SB600 (118,124), Ly6C-PE (clone AL-21), F4/80-PERCP (clone BM8), MHC-II-RB744 (clone M5/114), CCR2-AF467 (clone SA203G11), CX3CR1-BV650 (clone SA011F11), IL-17-BV510 (clone 2B8), and CD3-FITC (clone 17A2). Briefly, cells were selected based on CD45 expression and light scattering (FSC/SSC), followed by doublet and dead cell exclusion (using viability dye). Leukocytes were separated into lymphoid (CD3^+^) and myeloid (CD11b^+^) lineages. Key populations, including T cell subsets (CD4^+^, CD8^+^, γδ^+^), neutrophils (Ly6G^+^CX3CR1^–^), and monocyte/macrophage populations (Ly6C, F4/80, CX3CR1, CCR2, MHC-II), were then identified based on the strategy detailed in Figure S3.

### 5.12 Data Analysis

Unless stated otherwise, figures and statistical analyses were performed using the ggplot2 and Tidyverse packages in R version 4.4.3.^72^ Outliers were identified using Grubb’s test, and data normality was evaluated with the Shapiro–Wilk test. For normally distributed data, comparisons between two groups were performed using an unpaired Student’s *t*-test, whereas multiple-group comparisons were conducted via one-way or two-way ANOVA, followed by Tukey’s posthoc test. Non-normally distributed data were analyzed using the Mann–Whitney Utest (two groups) or the Kruskal–Wallis test (multiple groups). Survival curves were generated using the Kaplan–Meier method and compared with the log-rank test. Data are presented as mean ± SEM, and *p* values of ≤0.05 were considered statistically significant.

For high-dimensional flow cytometry analysis, dimensionality reduction was performed using the t-distributed stochastic neighbor embedding (t-SNE) algorithm, and cell clustering was performed using the Rphenograph algorithm within the Cytofkit2 R package.^73^

## Supplementary Material



## Notes

Supplemental data on parasite load, cardiac pathology, and electrocardiographic parameters; complete flow cytometry gating strategy for cardiac leukocytes; and comprehensive cytokine ratio analyses for cardiac T cell subsets, macrophages, neutrophils, and monocytes (PDF)
