# Supplementary material for: IL-33/ST2 Signaling Protects the Heart by Restraining Inflammation and Parasite Burden during Trypanosoma cruzi Experimental Infection
Source: ACS Infect Dis. 2025 Dec 9;12(1):342–62. doi: 10.1021/acsinfecdis.5c00859 (PMC12797244; doi:10.1021/acsinfecdis.5c00859)
Supplement: Supplementary file 1 [file id5c00859_si_001.pdf]

Supporting information

IL-33/ST2 signaling protects the heart by restraining inflammation and parasite burden during *Trypanosoma cruzi* experimental infection

Marcelo Eduardo Cardozo[1,2], Tatyane Martins Cirilo[1], Jorge Lucas Nascimento Souza[1], José Bryan da Rocha Rihs[1], Isabela de Brito Duval[1], Fernando Bento Rodrigues Oliveira[3], Mayra Ricci[3], Laura Lis de Oliveira Santos[3], Livia Fernanda Livia Fernanda Santana[3], Luiza Pinheiro Silva[3], Chiara Cássia Oliveira Amorim[1], Ana Rafaela Antunes-Porto[1], Izabela da Silva Oliveira[1], Ana Laura Grossi de Oliveira[1], Luisa Vitor Braga do Amaral[1], Gabriela Gomes Monteiro Lemos[1], Getúlio Mota e Silva Junior[1], Ivan Lobo de Sousa Marques[4], Marina Possa dos Reys[5], Geovanni Dantas Cassali[5], Artur Santos Miranda[4], Luisa Mourão Dias Magalhães[6], Lilian Lacerda Bueno[1,2], Fabiana Simão Machado[2,3*], Ricardo Toshio Fujiwara[1,2*].

[1] Laboratory of Immunobiology and Parasite Control, Institute of Biological Sciences, Universidade Federal de Minas Gerais, Belo Horizonte, Brazil,

[2] Post-graduation Program in Health Sciences: Infectious Diseases and Tropical Medicine, Faculdade de Medicina, Universidade Federal de Minas Gerais, Belo Horizonte, Brazil,

[3] Laboratory of Immunoregulation of Infectious Diseases, Institute of Biological Sciences, Universidade Federal de Minas Gerais, Belo Horizonte, Brazil,

[4] Laboratory of Cellular Electrophysiology, Institute of Biological Sciences, Institute of Biological Sciences, Universidade Federal de Minas Gerais, Belo Horizonte, Brazil,

[5] Laboratory of Comparative Pathology, Institute of Biological Sciences, Institute of Biological Sciences, Universidade Federal de Minas Gerais, Belo Horizonte, Brazil,

[6] Laboratory of Interactions in ImmunoParasitology, Institute of Biological Sciences, Institute of Biological Sciences, Universidade Federal de Minas Gerais, Belo Horizonte, Brazil

***Author for correspondence:** Ricardo Toshio Fujiwara, E-mail: rtfujiwara@gmail.com ; Fabiana Simão Machado, E-mail: machadofs@gmail.com

Table S1: Comparison of the mean values of heart parasite burden between WT and ST2$^{-/-}$ infected animals

**Parasite burden (log10 copies/ng of tissue)**		
Days post-infection	WT + Tc	ST2$^{-/-}$ + Tc
7 dpi	218.94	238.83
20 dpi	1862.37	3667.33
100 dpi	0.03	1.03

Table S2: Comparison of the mean values of EKG parameters between WT and ST2$^{-/-}$ infected animals

	WT NI	WT Tc	ST2$^{-/-}$ NI	ST2$^{-/-}$ + Tc
Heart rate (bpm)	370.06	405.49	348.93	421.28
PR segment (ms)	4.79	4.27	4.33	2.88

QRS interval (ms)	34.39	33.46	35.86	51.77
QRS axis (°)	32.02	53.19	38.19	-18
QT interval (ms)	67.66	56.39	68.66	74.88
QTc (ms)	621.48	668.73	574.29	696.44
Q amplitude (mV)	-0.01	-0.007	-0.02	-0.01
R amplitude (mV)	0.25	0.17	0.23	0.24
S amplitude (mV)	0.02	0.02	0.02	0.01
ST height (mV)	0.01	0.01	0.02	0.03
T amplitude (mV)	0.01	0.02	0.01	0.01

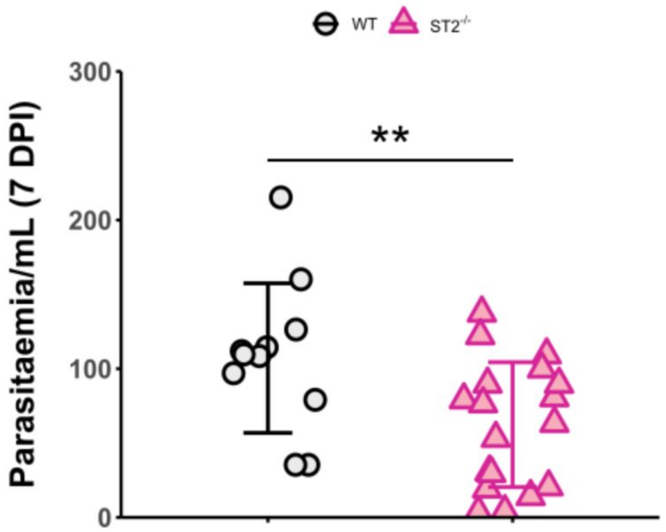

Figure S1. Blood parasitism at 7 dpi in *T. cruzi*-infected WT and ST2$^{-/-}$ mice. WT and ST2$^{-/-}$ mice were infected with 1000 *T. cruzi* blood trypomastigotes. Data show the number of parasites per mL of blood. Results are represented by the mean ± SD of two independent experiments. **p < 0.01. Data were analyzed using t test. WT: wild-type.

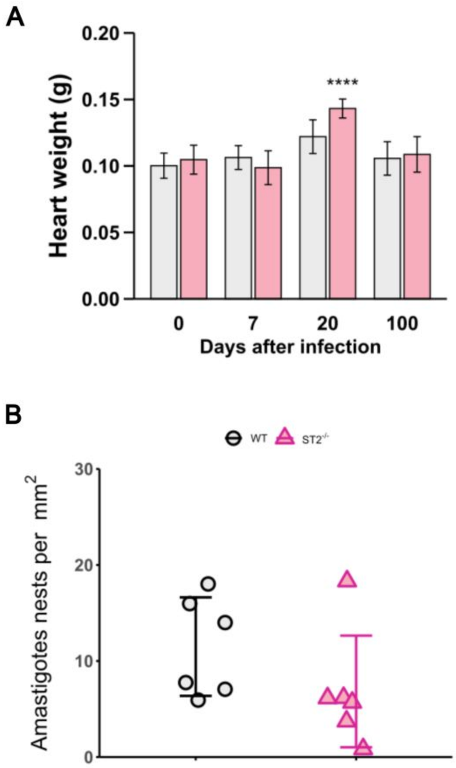

Figure S2. Heart weight and heart amastigote nests during *T. cruzi* infection in WT and ST2$^{-/-}$ mice. WT and ST2$^{-/-}$ mice were infected with 1000 *T. cruzi* blood trypomastigotes and monitored for up to 100 days, covering both acute and chronic phases of infection. (A) Heart weight (g). (B) Amastigote nest counts in heart tissues at 20 dpi. Results are represented by the mean ± SD. ****p < 0.0001. Asterisks represent comparison between each strain and its respective uninfected group (0 dpi). Asterisks with bars represent comparison between strains at the same time point. Time series data were analyzed using Two-way ANOVA with Tukey's post hoc test (A) or t test (B). WT: wild-type.

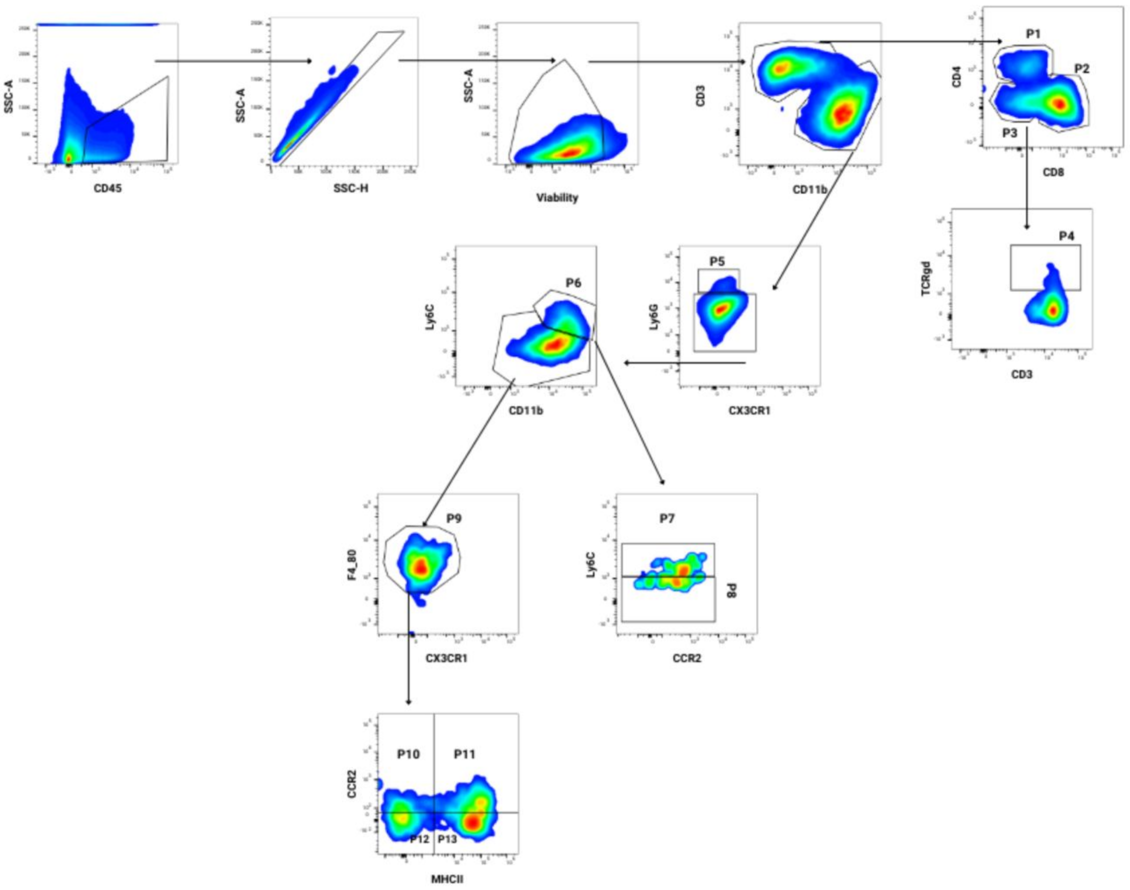

Figure S3. Cardiac cell population gating strategy. Cardiac cell population analysis strategy, in which cells were sequentially selected based on CD45 expression and light scattering parameters (FSC/SSC), followed by doublet discrimination and selection of viable leukocytes. From this population, cells were separated into lymphoid ($CD3^{+}$) and myeloid ($CD11b^{+}$) lineages. In the lymphoid lineage, helper T lymphocytes (P1: $CD4^{+}$), cytotoxic T lymphocytes (P2: $CD8^{+}$), double-negative T lymphocytes (P3: $CD4^{-}CD8^{-}$), and gamma-delta T cells (P4: $TCR\gamma\delta^{+}$) were identified. To characterize the myeloid lineage, neutrophils (P5: $Ly6G^{+}CX3CR1^{-}$) were first identified from the $CD11b^{+}$ cell gate. The remaining population ($CD11b^{+}Ly6G^{-}$) was then used for parallel analyses of monocytes and macrophages. Monocytes were identified as $Ly6C^{+}$ cells (P6) and subsequently divided into classical (P7: $Ly6C^{high}$ $CCR2^{+}$) and non-classical (P8: $Ly6C^{low}$ $CCR2^{-}$). From the same $CD11b^{+}Ly6G^{-}$ population, macrophages (P9:

F4/80⁺CX3CR1⁺) were identified and then sub-clustered based on MHCII and CCR2 expression (populations P10 to P13).

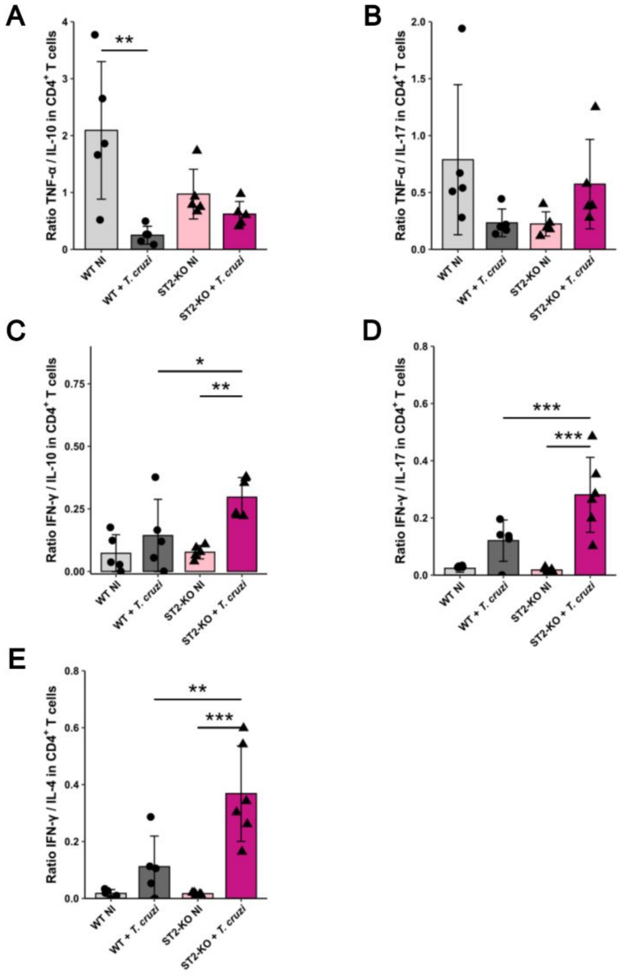

Figure S4. Analysis of CD4+ T cell cytokine ratios in heart tissue by flow cytometry in WT and $ST2^{-/-}$ mice infected with *T. cruzi* at 20 dpi. (A) Assessment of TNF/IL-10 ratio in CD4+ T cells. (B) TNF/IL-17 ratio in CD4+ T cells. (C) IFN-γ/IL-10 ratio in CD4+ T cells. (D) IFN-γ/IL-17 ratio in CD4+ T cells. (E) IFN-γ/IL-4 ratio in CD4+ T cells. Statistical analyses were conducted between each strain and its uninfected group and between the two strains at the same infection time. N = 5-6 biological replicates per group. Results are represented by the mean ± SD. *$p < 0.05$, **$p < 0.01$, ***$p < 0.001$,

****p < 0.0001. Data were analyzed using One-way ANOVA with Tukey's post hoc test. WT: wild-type.

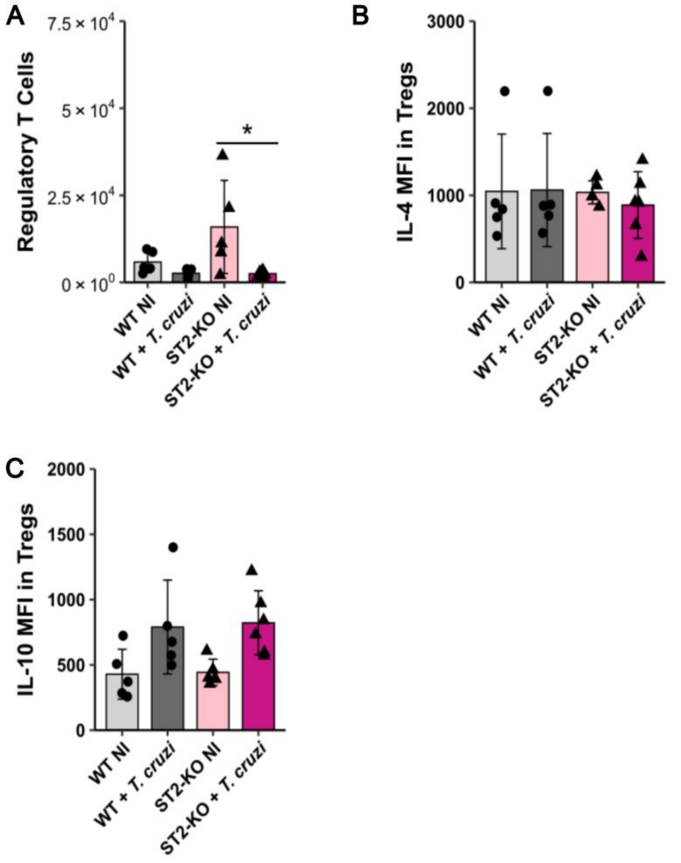

Figure S5. Analysis of Regulatory T cells in heart tissue by flow cytometry in WT and ST2$^{-/-}$ mice infected with *T. cruzi* at 20 dpi. (A) Total number of regulatory T cells. (B) Expression intensity of IL-4 in regulatory T cells. (C) Expression intensity of IL-10 in regulatory T cells. Statistical analyses were conducted between each strain and its uninfected group and between the two strains at the same infection time. N = 5-6 biological replicates per group. Results are represented by the mean ± SD. *p < 0.05. Data were analyzed using One-way ANOVA with Tukey's post hoc test. WT: wild-type.

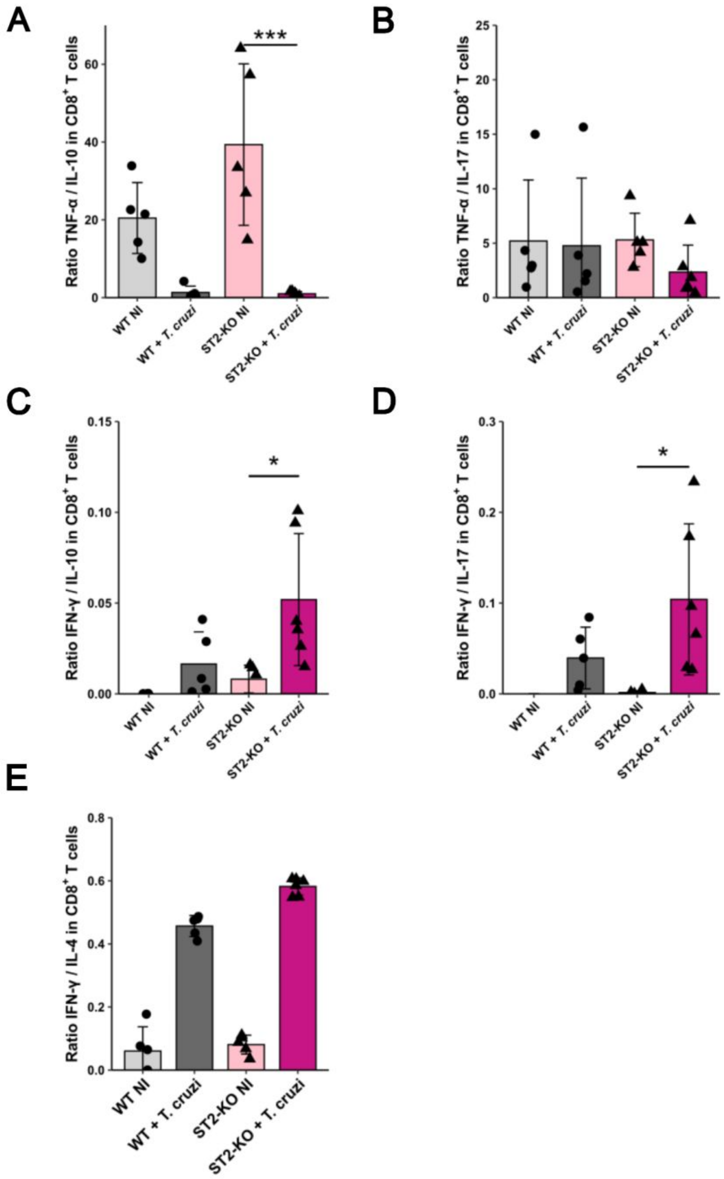

Figure S6. Analysis of CD8+ T cell cytokine ratios in heart tissue by flow cytometry in WT and ST2$^{-/-}$ mice infected with *T. cruzi* at 20 dpi. (A) Assessment of TNF/IL-10 ratio in CD8+ T cells. (B) TNF/IL-17 ratio in CD8+ T cells. (C) IFN-γ/IL-10 ratio in CD8+ T cells. (D) IFN-γ/IL-17 ratio in CD8+ T cells. (E) IFN-γ/IL-4 ratio in CD8+ T cells. Statistical analyses were conducted between each strain and its uninfected group and between the two strains at the same infection time. N = 5-6 biological replicates per group. Results are represented by the mean ± SD. *p < 0.05, ***p < 0.001. Data were analyzed using One-way ANOVA with Tukey's post hoc test. WT: wild-type.

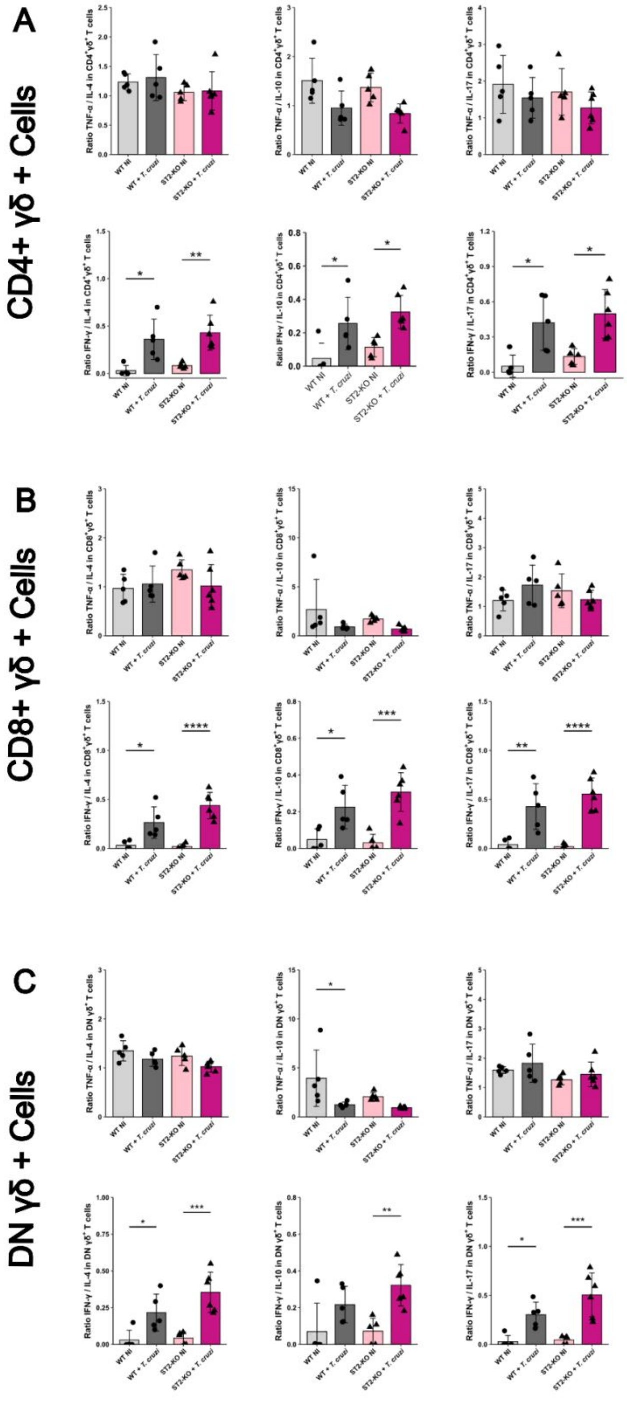

Figure S7. Analysis of γδ+ T cell cytokines ratios in heart tissue by flow cytometry in WT and ST2$^{-/-}$ mice infected with *T. cruzi* at 20 dpi. (A) Assessment of TNF/IL-4, TNF/IL-10, TNF/IL-17, IFN-γ/IL-4, IFN-γ/IL-10, and IFN-γ/IL-17 ratios in CD4+ γδ+ T cells. (B) TNF/IL-4, TNF/IL-10, TNF/IL-17, IFN-γ/IL-4, IFN-γ/IL-10, and IFN-γ/IL-17

ratios in CD8+ γδ+ T cells. (C) TNF/IL-4, TNF/IL-10, TNF/IL-17, IFN-γ/IL-4, IFN-γ/IL-10, and IFN-γ/IL-17 ratios in double-negative (DN) γδ+ T cells. Statistical analyses were conducted between each strain and its uninfected group and between the two strains at the same infection time. N = 5-6 biological replicates per group. Results are represented by the mean ± SD. $*p < 0.05$, $**p < 0.01$, $***p < 0.001$, $****p < 0.0001$. Data were analyzed using One-way ANOVA with Tukey's post hoc test. WT: wild-type.

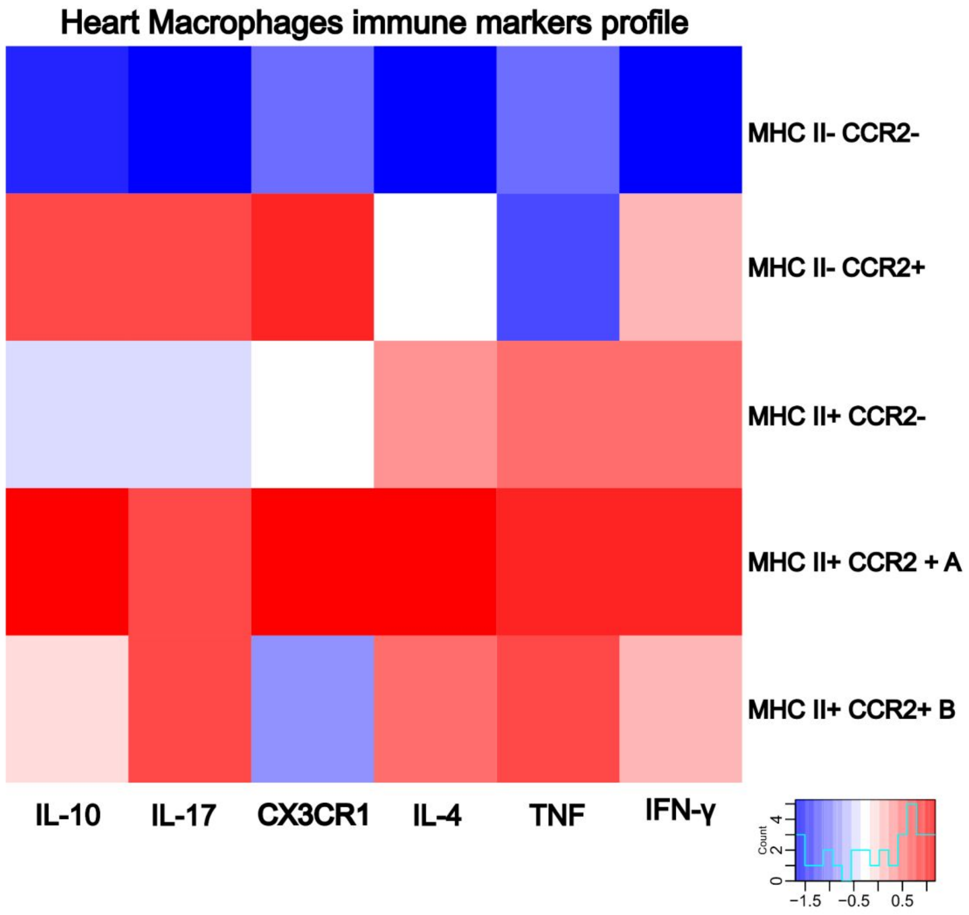

Figure S8. Heatmap of immune marker expression in different heart macrophage (HM) subsets. The profiles of MHC II, CCR2, and CX3CR1 were used to determine HM subsets in heart tissue by flow cytometry in WT and ST2$^{-/-}$ mice infected with *T. cruzi* at 20 dpi. Expression profiles of cytokines (IL-10, IL-17, IL-4, TNF, IFN-γ) and the chemokine receptor CX3CR1 were evaluated in different HM subsets.

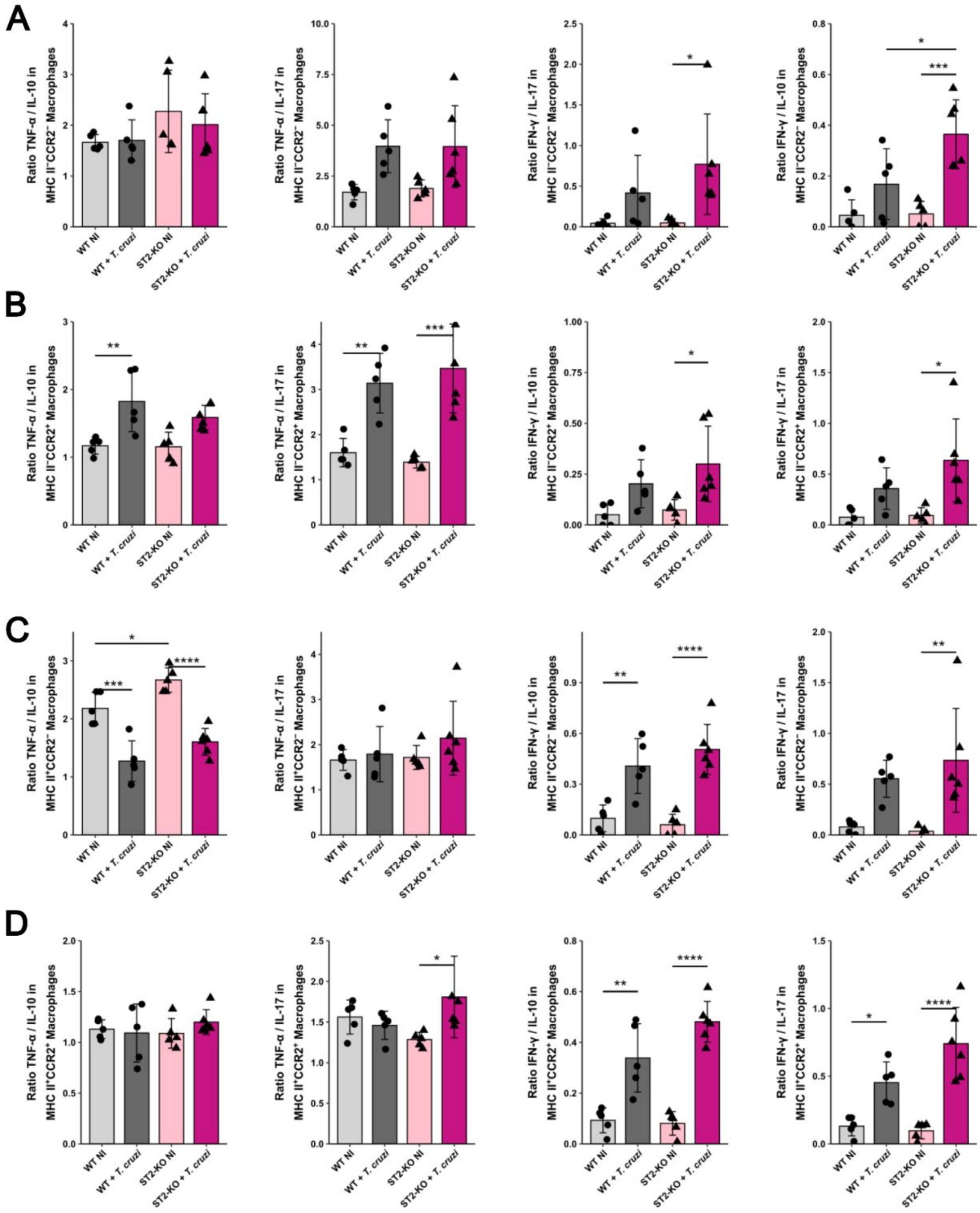

Figure S9. Analysis of heart macrophages (HMs) cytokine ratios in heart tissue by flow cytometry in WT and ST2$^{-/-}$ mice infected with *T. cruzi* at 20 dpi. (A) Assessment of TNF, IL-10, IL-17 and IFN-γ ratios in MHC II- CCR2- HMs. (B) TNF, IL-10, IL-17 and IFN-γ ratios in MHC II- CCR2+ HMs. (C) TNF, IL-10, IL-17 and IFN-γ ratios in MHC II+ CCR2- HMs. (D) TNF, IL-10, IL-17 and IFN-γ ratios in MHC II+ CCR2+ HMs. Statistical analyses were conducted between each strain and its uninfected group

and between the two strains at the same infection time. N = 5-6 biological replicates per group. Results are represented by the mean ± SD. *p < 0.05, **p < 0.01, ***p < 0.001, ****p < 0.0001. Data were analyzed using One-way ANOVA with Tukey's post hoc test. WT: wild-type.

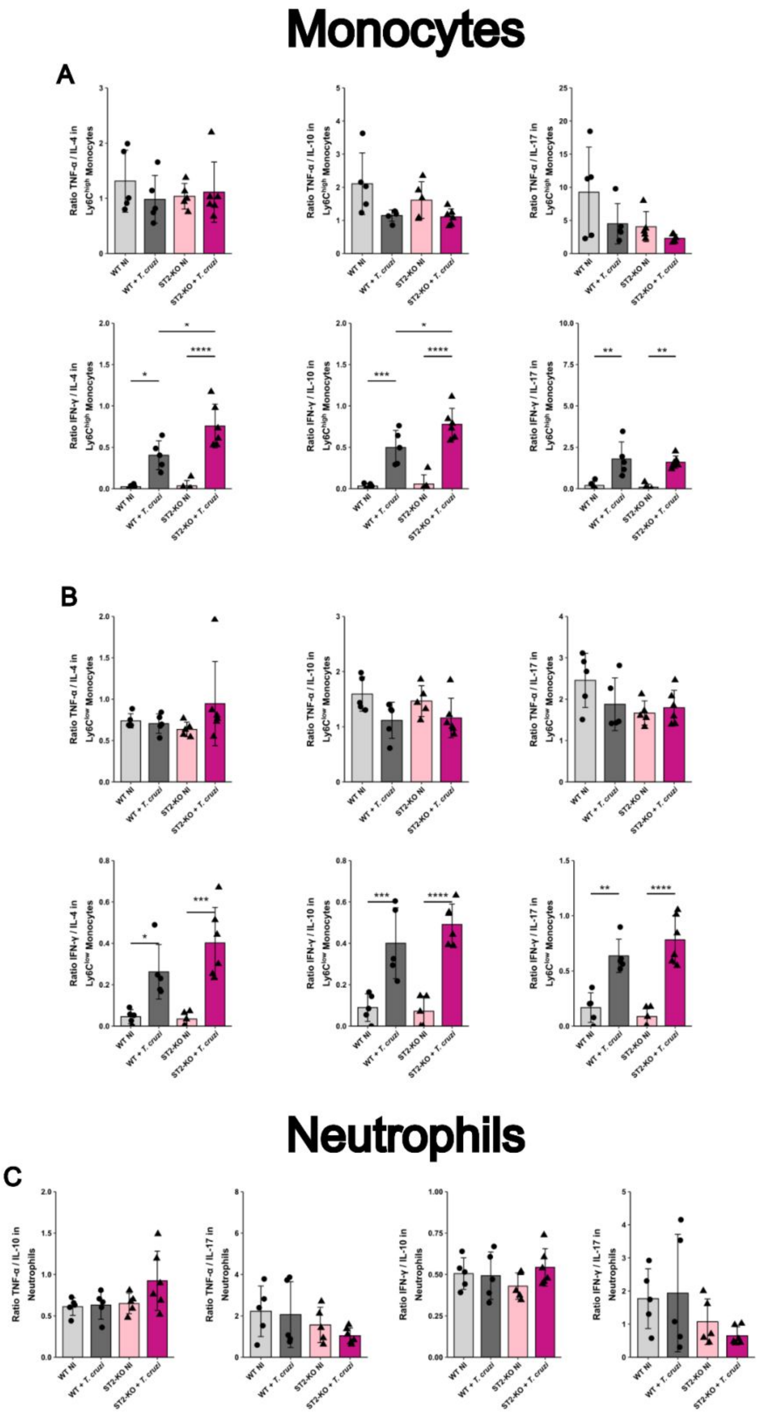

Figure S10. Analysis of monocytes and neutrophil cytokine ratios in heart tissue by flow cytometry in WT and ST2$^{-/-}$ mice infected with *T. cruzi* at 20 dpi. (A) Assessment of TNF, IL-4, IL-10, IL-17 and IFN-γ ratios in Ly6C^{high} monocytes. (B) TNF, IL-4, IL-10, IL-17 and IFN-γ ratios in Ly6C^{low} monocytes. (C) TNF, IL-10, IL-17 and IFN-γ ratios in neutrophils. Statistical analyses were conducted between each strain and its uninfected group and between the two strains at the same infection time. N = 5-6 biological replicates per group. Results are represented by the mean ± SD. *$p < 0.05$, **$p < 0.01$, ***$p < 0.001$, ****$p < 0.0001$. Data were analyzed using One-way ANOVA with Tukey's post hoc test. WT: wild-type.
